# 2-Methyl-4-chlorophenoxyacetic acid (MCPA) sorption and desorption as a function of biochar properties and pyrolysis temperature

**DOI:** 10.1371/journal.pone.0291398

**Published:** 2023-09-08

**Authors:** Abdullah Niaz, Kurt A. Spokas, Bea Gámiz, David Mulla, Khaliq R. Arshad, Sarfraz Hussain

**Affiliations:** 1 Pesticide Residue Laboratory, Institute of Soil Chemistry & Environmental Sciences, Kala Shah Kaku, Punjab, Pakistan; 2 Department of Soil, Water and Climate, University of Minnesota, St. Paul, MN, United States of America; 3 United States Department of Agriculture, Agricultural Research Service, St. Paul, MN, United States of America; 4 Department of Inorganic Chemistry, Chemical Institute for Energy and the Environment (IQUEMA), University of Córdoba, Córdoba, Spain; University of South Africa, SOUTH AFRICA

## Abstract

2-Methyl-4-chlorophenoxyacetic acid (MCPA) is a highly mobile herbicide that is frequently detected in global potable water sources. One potential mitigation strategy is the sorption on biochar to limit harm to unidentified targets. However, irreversible sorption could restrict bioefficacy thereby compromising its usefulness as a vital crop herbicide. This research evaluated the effect of pyrolysis temperatures (350, 500 and 800°C) on three feedstocks; poultry manure, rice hulls and wood pellets, particularly to examine effects on the magnitude and reversibility of MCPA sorption. Sorption increased with pyrolysis temperature from 350 to 800°C. Sorption and desorption coefficients were strongly corelated with each other (R^2^ = 0.99; P < .05). Poultry manure and rice hulls pyrolyzed at 800°C exhibited irreversible sorption while for wood pellets at 800°C desorption was concentration dependent. At higher concentrations some desorption was observed (36% at 50 ppm) but was reduced at lower concentrations (1–3% at < 5 ppm). Desorption decreased with increasing pyrolysis temperature. Sorption data were analyzed with Langmuir, Freundlich, Dubinin–Radushkevich and Temkin isotherm models. Freundlich isotherms were better predictors of MCPA sorption (R^2^ ranging from 0.78 to 0.99). Poultry manure and rice hulls when pyrolyzed at higher temperatures (500 and 800°C) could be used for remediation efforts (such as spills or water filtration), due to the lack of desorption observed. On the other hand, un-pyrolyzed feedstocks or biochars created at 350°C could perform superior for direct field applications to limit indirect losses including runoff and leaching, since these materials also possess the ability to release MCPA subsequently to potentially allow herbicidal action.

## Introduction

Extensive pesticide use has severely affected the quality of soil, water and air, bringing undesirable impacts, including toxicity and carcinogenicity, on non-target species including humans [1]. Pesticide contamination could negatively affect soil quality, nutrient cycling, enzyme activity, soil biota, biodiversity and endangering other ecosystem processes [2]. There is always a potential risk of groundwater pollution and pesticides being the most intensively observed chemicals in European groundwater [3]. Recent research has shown pesticide residues in 60% of studied groundwater samples, from both urban and agricultural farms in the United States [4]. 2-Methyl-4-chlorophenoxyacetic acid (MCPA) is a hormone-type, selective, systemic herbicide, readily absorbed by roots and leaves, to be used for controlling annual as well as perennial weeds in grassland, cereals and turf [5]. Additionally, MCPA is used to control aquatic broad leaf weeds [6]. MCPA is a weak organic carboxylic acid with a pK_a_ value of 3.1, meaning that it will predominantly be in its anionic form at typical soil pH values (between 5 to 8) [7]. More than 2,000 t of MCPA are used only in Western Europe each year [8]. MCPA is highly mobile in soils, with K_oc_ from 50 to 62 L kg^-1^ and has a short to moderate 7 to 41 day half-life [9,10]. These two factors suggest that MCPA has a potential risk for contaminating potable water sources [11,12]. In fact, MCPA is one of the most widespread pesticides detected globally in lakes, rivers, and groundwater [13–15].

Biochar, a carbon-enriched product, comes from the thermal decomposition of organic material, including animal manure [16], crop residues [17], woody materials [18], biosolids [19], industrial by-products, urban-yard wastes and sewage sludge [20]. These carbon-rich materials due to biomass pyrolysis have been reported to increase crop yields through enhanced water-holding capacity and nutrient availability [21,22] and are a means of sequestering carbon in soil [23]. Sorption of organic and inorganic pollutants by biochar is due to aromaticity and higher surface area [24], large pore structure, diverse surface functional groups, high pH, and high cation exchange capacity [25]. Several studies have established sorption of various pesticides on biochar [18,26] and showing biochar can be a “super-sorbent” for contaminants [27]. Sorption on biochar could have numerous benefits, including, reducing leaching and runoff losses [28]. However, at the same time biochar can retain pesticides in soils for longer periods due to retarding pesticide dissipation [29,30] and protecting pesticides from microbial mineralization [28,31].

Another important environmental issue worldwide could be agricultural wastes being produced globally in millions of tons every year [32]. The worldwide burning of annually generated crop residues could cause very large environmental risk, producing atmospheric pollution and significant carbon particulates. In India alone, the total estimated annual crop residues left-over is about 141 Mt, out of this 93 Mt crop residues were burnt on-farm [33]. Crop residues could be low-cost feedstocks for biochar and an alternative for managing agricultural waste. This approach is quite sustainable and economically feasible for converting wastes into biochar [34]. Studies have shown that biochars from wood, wheat and rice residues could be 400–2500 times more effective than the original soil for pesticide sorption [35].

Sorption capacity of biochar might not be as high as other types of sorbents, like activated carbon or even clays (i.e., smectite) [36,37]. So, modifications of biochar, to enhance its sorption capacity, have also been reported, including loading organic functional groups and alkali activation [38,39], mineral loading on biochar such as magnetite, hematite, manganese oxide, zero valent Fe, and calcium oxide [28]. Additionally, studies have incorporated nanoparticles, such as nanocrystals of ZnS [40] or loading 1% graphene on the biochar surface [41]. However, if these modified biochars are applied to soil, it is important to consider not only economic viability, but also the need to evaluate potential contamination by the various activating agents [28]. Contrary to other comparatively complex ways for increased pesticide sorption, merely selecting an appropriate feedstock and variation in pyrolysis temperature during biochar preparation could optimize pesticide sorption on biochar surfaces [42–45]. It has been established that biochar application effects in soils depend mainly on the feedstock and pyrolysis temperature [46,47].

Longer persistence and reduced mobility of pesticides due to sorption on biochar could reduce the efficacy of soil-applied pesticides, especially that of herbicides [48], making weed control more problematic [49]. Therefore, there are two aspects of pesticide sorption on biochar. On one side, it might be considered favorable, thereby reducing the leaching, bioavailability and the non-target toxicity due to pesticides in soil [50]. While on the other, reduced bioavailability can be problematic in an agricultural setting, since it can reduce the plant uptake and efficacy of pre-emergent herbicides, requiring the farmer to apply more pesticides/herbicides on a biochar-amended field [51].

Therefore, better knowledge about locally available feedstocks and how various pyrolysis temperatures affect adsorption-desorption properties are needed to guide selection of biochar properties to improve adsorption to protect the environment, while allowing some desorption back to the soil solution, thereby maintaining good bioefficacy for weed control. The present study aimed to characterize and compare biochars, for MCPA sorption and desorption abilities. Three biomass residues were studied; poultry manure, rice hulls and wood pellets, subjected to three pyrolysis temperatures (350, 500 and 800°C). The hypothesis is that MCPA sorption and desorption potentials can be optimized by specific combinations of feedstock and pyrolysis temperature.

## Materials and methods

### Chemicals

Radiolabeled ^14^C material of 4-chloro-2 methylphenoxy acetic acid sodium salt monohydrate (MCPA; CAS [3653-48-3]) was obtained from Moravek, Inc. (MC-2515; Brea, CA USA). Radiolabeled MCPA [ring-U-^14^C] had a specific activity of 12.3 MBq mg^-1^. Additionally, non-labelled MCPA was obtained from ChemService, Inc. (N-10818-250MG; PA, USA) and reagent grade (99% purity) of CaCl_2_ dihydrate was obtained from Sigma-Aldrich (223506; St. Louis, MO USA). A summary of the key properties of MCPA is given in Table 1.

**Table 1:** Key properties of MCPA.

Characteristics	Value
Density 20 °C (g cm^−3^)	1.56
Solubility 20 °C (mg L^−1^)	270,000
Negative logarithm of the acid dissociation constant (pK_a_ = -log K_a_)	3.1 [7]
Soil half-live (t_½_; days)	7–41 [9,10]
Soil sorption coefficient (K_D;_ L kg^-1^)	0.3–1.5 [52–55]

### Biochars

Three different feedstocks [poultry manure (PM), rice husks (RH) and wood pallets (WP)] were used to prepare the biochars for evaluation. These were coded with the two-letter feedstock followed by pyrolysis temperature in°C or RAW in subscript designating the original non-pyrolyzed feedstock. A total of 12 different sorbents, including raw form and biochars created at three pyrolysis temperatures (350, 500 and 800°C) were named as; PM_RAW_, PM350, PM500, PM800, RH_RAW_ RH350, RH500, RH800, WP_RAW,_ WP350, WP500 and WP800. Prior to pyrolysis, the feedstocks were oven dried for 24 h at 60°C, followed by mechanical sieving to < 4 cm. The feedstocks were pyrolyzed using a box furnace-retort system (Lindberg/MPH, Riverside, MI), between 0.5 and 1.5 kg of ground material was loaded onto a stainless-steel tray or into a crucible and placed into a gas tight retort for 2 h under a stream of N_2_ gas at the desired pyrolytic temperature. The samples were stored in sealed low density polyethylene film (LDPE) bags (Ziploc^TM^) until use (< 6 months from the time of biochar production).

### Characterization of biochars

Loss on drying was determined in air at 105°C overnight and is reported on an as received sample basis. All other results are reported on a dried sample basis. Elemental (C, H, N, and O) analysis was carried out using thermal combustion (Hazen Research, Golden, CO USA). Ash percentage was determined after heating to 750°C in air and holding at temperature for 8 h. Molar ratios of C:N, H:C, and O:C were also calculated from the elemental composition data. The Fourier-transform infrared (FTIR) spectroscopy data were collected using the attenuated total reflection (ATR; ThermoFisher iS50 FTIR spectrometer). The ATR used a diamond crystal. In the data collection, the resolution was 2 cm^-1^ and the number of scans for each spectrum was 32. The averaged spectrum from each sample was then displayed on an absorbance scale. Surface analyses were performed by nitrogen sorption analysis (MSE Analytical Services, Tucson, AZ, USA). Samples were degassed at 250°C for 8 h prior to analysis. Surface areas were estimated at -196°C using N_2_ and the Brunauer–Emmett–Teller method, based on the best linear fit for points in the 0.025–0.30 P/P_0_ range [56]. Total pore volume was estimated through the Barrett-Joyner-Halenda (BJH) method on the N_2_ absorption data [57]. Scanning electron micrographs were also collected for the biochars and feedstocks. The physico-chemical properties of studied biochars were also correlated with MCPA sorption and desorption K_D_ to assess for any significant relationships.

### Sorption study

Sorption was measured by laboratory batch sorption experiments, according to modified OECD 106-Tier 3 guidelines (OECD, 2000). Each sorbent (0.25 g dry weight) was mixed with different volume combinations of 0.01 M CaCl_2_ and unlabeled MCPA (S1 Table in S1 Text) followed by a fixed volume of ^14^C-labelled MCPA, to achieve multiple initial concentrations of 0.1, 0.9, 4.7, 9.4, 25 and 50 μg MCPA mL^-1^, which corresponds to 450,000 Bq per vial (8,440 DPM mL^-1^) over the range of MCPA concentrations. These sorption experiments were conducted in triplicate.

Each sorbent was placed in 20-mL glass scintillation vials, followed by the corresponding liquid injections from S1 Table in S1 Text, which were then placed horizontally on a reciprocal shaker and allowed to equilibrate for 24 h (180 rev min^-1^) in the dark. Following this period, the samples were centrifuged (20 min, 1,500 × g), a sample of supernatant (1 mL) was removed and filtered through 0.45 μm syringe filter. Then a 0.20-mL aliquot of the filtered solution was mixed with 6 mL of scintillation cocktail [EcoLite(+); MP Biomedicals, LLC] and analyzed for ^14^C by liquid scintillation counting (LSC) (HITACHI AccuFLEX LSC-8000, GMI Ramsey, MN; 10-min counting window). No statistically significant sorption of MCPA to scintillation vials, syringes, pipette tips, or syringe filters was observed (88–100% recovery; data not shown). Additionally, previous studies with MCPA have not detected significant degradation in laboratory incubations in 24 h [58]. The MCPA sorbed concentration on biochar (C_s_; μg g^-1^) was estimated by the following:

$$C_{S}=\frac{(C_{i}-C_{e})V}{m},$$

where C_i_ is the initial MCPA concentration (μg mL^-1^), C_e_ is the equilibrium MCPA concentration (μg mL^-1^), V is the total volume of the liquid phase (mL), and m is the mass of the biochar (g). The liquid concentration was determined by the LSC of the liquid phase using the following equation:

$$C_{e}=\frac{({LSC}_{e}-Blank)C_{i}}{{LSC}_{i}},$$

where LSC_e_ is the disintegrations per minute of the sample following equilibration with the biochar, Blank is the disintegrations per minute (DPM) of the scintillation cocktail and vial alone, C_i_ is the liquid phase concentration of the initial standard (μg mL^-1^), and LSC_i_ is the blank corrected DPM of the corresponding MCPA standards (without biochar; 8,440 DPM mL^-1^).

Average liquid (C_e_) and solid (C_s_) concentrations were calculated from the three replicates and then analyzed by fitting to four different sorption models: Langmuir, Freundlich, Temkin, and the Dubinin–Radushkevich models by the R package *PUPAIM* [59]. The individual isotherm models are given in (S2 Table in S1 Text). All isotherm models were fitted using nonlinear regression curve fitting in R using the PUPAIM package [59]. An example R script for performing the isotherm fitting is provided in the Supplemental Information, including the graphical output (see S2 Text). The Langmuir isotherm is based on monolayer sorption to a fixed number of homogeneous sorption sites, with the maximum sorption capacity estimated by the coefficient Q_m_. The value of the Freundlich coefficient $\frac{1}{n}$ provides insight into the distribution of sorption site heterogeneity with values closer to zero indicating higher degree of site heterogeneity. Although not theoretically derived, $\frac{1}{n}$ values of close to zero have been attributed to chemisorption and greater than one to more cooperative sorption mechanisms [60,61], meaning that the presence of adsorbates on the absorbent’s surface enhances the adsorption processes. Additionally, the $\frac{1}{n}$ values have been correlated with the physio-chemical properties of the adsorbent [62]. The Temkin model expects indirect adsorbate/adsorbate interactions on the adsorption process, and provides an estimated heat of adsorption through the value of its coefficient B_t_ [63]. The Dubinin–Radushkevich isotherm is an empirical relationship focused on a pore filling mechanism and has been applied to sorption by microporous materials [64]. An estimated mean free energy of sorption (E; J mol^-1^) can be estimated from the coefficient K_DR_ with the following relationship [64]:

$$E=\frac{1}{\sqrt{2K_{DR}}}.$$

### Desorption study

A 5-mL aliquot of supernatant was removed after the first 24-h equilibration period and the initial adsorption was determined. Desorption of MCPA was accomplished by replacing the 5-mL aliquot removed from the equilibrated vial with 5 mL of fresh 0.01 M CaCl_2_, at each concentration tested for sorption. The vials were vortexed to disperse the biochar sample back into the solution, mechanically shaken as previously described for 24 h, and then recentrifuged. The concentration of MCPA present in the aqueous solution after the 24-h desorption period was determined by LSC, as previously described, except for using 0.5 mL of the filtered supernatant for LSC counting. The desorption isotherm was fitted to the Freundlich model and compared to the original sorption isotherms. Specifically, the hysteresis (H) for MCPA sorption was quantified using formula using the Freundlich isotherm coefficients [65]:

$$H=\frac{{\left(\frac{1}{n}\right)}_{desorption}}{{\left(\frac{1}{n}\right)}_{sorption}}.$$

The H coefficient has values between 0 for a completely irreversible processes and 1 when the sorption isotherm follows the same pathway as sorption (reversible).

### Statistical analysis

All data for the K_D_ determination were conducted in triplicate, with means and standard deviations of the measurements presented. All statistical analyses were performed in R [66]. The data for the isotherm analysis were analyzed for each biochar using the R package *PUPAIM* [59], which calculates the corresponding model isotherm coefficients as well as the root mean square error (RMSE), mean absolute error (MAE), Akaike Information Criterion (AIC), Bayesian Information Criterion (BIC), and the corresponding Pearson’s correlation (R^2^) of the model fits. The model with the lowest overall AIC, BIC, MAE, and RMSE over all the samples was correspondingly selected as the best-fitting isotherm model for the data. Correlation analysis was performed utilizing the R package *ggcorrplot* [67] to evaluate relationships between the soil chemical and physical properties and the resulting sorption coefficients. Two-way ANOVA and Tukey’s HSD (Honestly Significant Difference) tests were used to compare K_D_ as a function of feedstock type and pyrolysis temperatures with the *agricolae* package [68] in R.

## Results and discussion

### Examination of biochar properties

The physical, chemical, and elemental properties of the biochars and original feedstocks are presented in Table 2. The observed air-dried moisture content of the raw feedstock samples showed poultry manure being the highest (7.3%) followed by rice hulls (5.5%) and wood pellets (4.3%). Higher pyrolysis temperatures reduced the air-dried moisture content, which was ascribed to the pyrolysis process itself altering the pore-structure of the various feedstocks. The volatilization of organic matter, water loss during dehydration, and collapse as well as fracturing during pyrolysis give birth to differing pore distributions in biochar as compared to the original feedstock [69]. As seen in Table 2, increasing pyrolysis temperature enhanced surface area as well as the estimated total pore volume. Although these increases were the greatest in the PM and WP feedstocks (Table 2). Pyrolysis processes are often complicated by the presence of various inorganic species [70], which could be an important facet determining the pyrolysis reactions for poultry manure and rice hulls.

**Table 2:** Elemental analysis and physico-chemical properties of feedstocks and biochars.

Biochar	Drying Loss	C	H	N	S	Ash	O	Volatile Matter	Fixed C	Molar Ratios					
										C:N		H:C	O:C	SSA	Pore Volume	pH
	% (w/w)												(m^2^ g^-1^)	(cm^3^ g^-1^)	
**PM**_**Raw**_	7.32	36.48	4.92	2.93	1.33	24	37.1	61.75	14.26	14.5	1.62	0.76	0.1	0.001	6.1
**PM350**	2.04	39.58	2.76	3.27	1.63	47.6	19.9	28.54	23.82	14.1	0.84	0.38	3.3	0.001	6.2
**PM500**	1.92	35.84	1.28	2.45	2.17	61.9	14.7	17.73	20.41	17.1	0.43	0.31	5.1	0.002	7.4
**PM800**	4.15	41.55	0.71	1.8	1.96	66.4	12.2	9.13	24.5	26.9	0.21	0.22	61.6	0.025	9.4
**RH**_**Raw**_	5.48	41.4	5.13	0.4	0.04	17.1	39.8	65.9	17.05	120.8	1.49	0.72	0.4	0.001	7.6
**RH350**	2.39	49.9	3.04	0.63	0.03	32	20.5	27.69	40.32	92.4	0.73	0.31	5.3	0.002	7.2
**RH500**	1.93	51.7	1.93	0.6	0.02	39.3	19.5	12.32	48.39	100.5	0.45	0.28	18.0	0.007	7.1
**RH800**	1.9	53.35	0.66	0.56	0.25	43.8	13.9	4.69	51.55	111.1	0.15	0.20	2.3	0.004	7.9
**WP**_**Raw**_	4.26	50.36	6.14	0.07	0.01	0.37	46.3	82.42	17.21	839.3	1.46	0.69	0.1	0.003	5.5
**WP350**	2.46	76.39	3.8	0.18	0.01	1.01	20.4	32.9	66.09	495.1	0.60	0.20	0.6	0.003	5.4
**WP500**	1.76	89.12	2.82	0.18	0.01	1.34	8.72	15.15	83.51	577.6	0.38	0.07	0.2	0.004	5.8
**WP800**	0.91	95.66	0.85	0.37	0.1	1.67	2.51	4.2	94.12	301.6	0.11	0.02	46.6	0.018	6.5

Notes: PM = Poultry manure; RH = Rice hulls; WP = Wood pellets; Subscripted RAW = Raw and unpyrolysed feedstocks; Numerical suffixes = Pyrolysis temperatures at 350°C, 500°C and 800°C. pH was determined by placing 0.25 g of each material in 3.2 mL of 0.01 M CaCl_2_ then equilibrated for 24 hr. SSA is the specific surface area.

Samples of biochars from PM and RH possessed higher ash content, due to higher amounts of inorganic species in the ash, which would result in oxygen being included in both the direct oxygen analysis and ash analysis results. The elemental analysis (C, H, N, S, and O) determined that WP contained higher C (total and fixed) than other samples (P<0.05). Carbon content typically increased with pyrolysis temperatures, although not statistically significant across all temperatures and feedstocks (P>0.05). The increased carbon contents at higher temperatures have been documented in prior studies [e.g., 44]. Heitkötter and Marschner [71] observed higher carbon contents at 600°C pyrolysis temperature than 400°C and importantly, the effect of heating was more pronounced in case of woody, pine chip-based biochars than in corn-based biochars. This was also observed in our study, where carbon contents increased more dramatically for wood pellet biochars compared to other two feedstocks: PM and RH. There was a strong correlation between C content and fixed carbon (R = 0.955; P<0.01).

Poultry manure showed higher N (2.9%) among the feedstocks which decreased with increases in pyrolysis temperature, suggesting a loss of volatile N compounds with the increasing pyrolysis temperatures. On the other hand, for two other materials, rice hulls and wood pellets, N contents increased slightly with increasing temperature, suggesting the N was conserved during the pyrolysis process with the loss of more volatile constituents. Overall, there was a correlation between N and S contents (R = 0.885; P<0.01).

**Fig 1:**
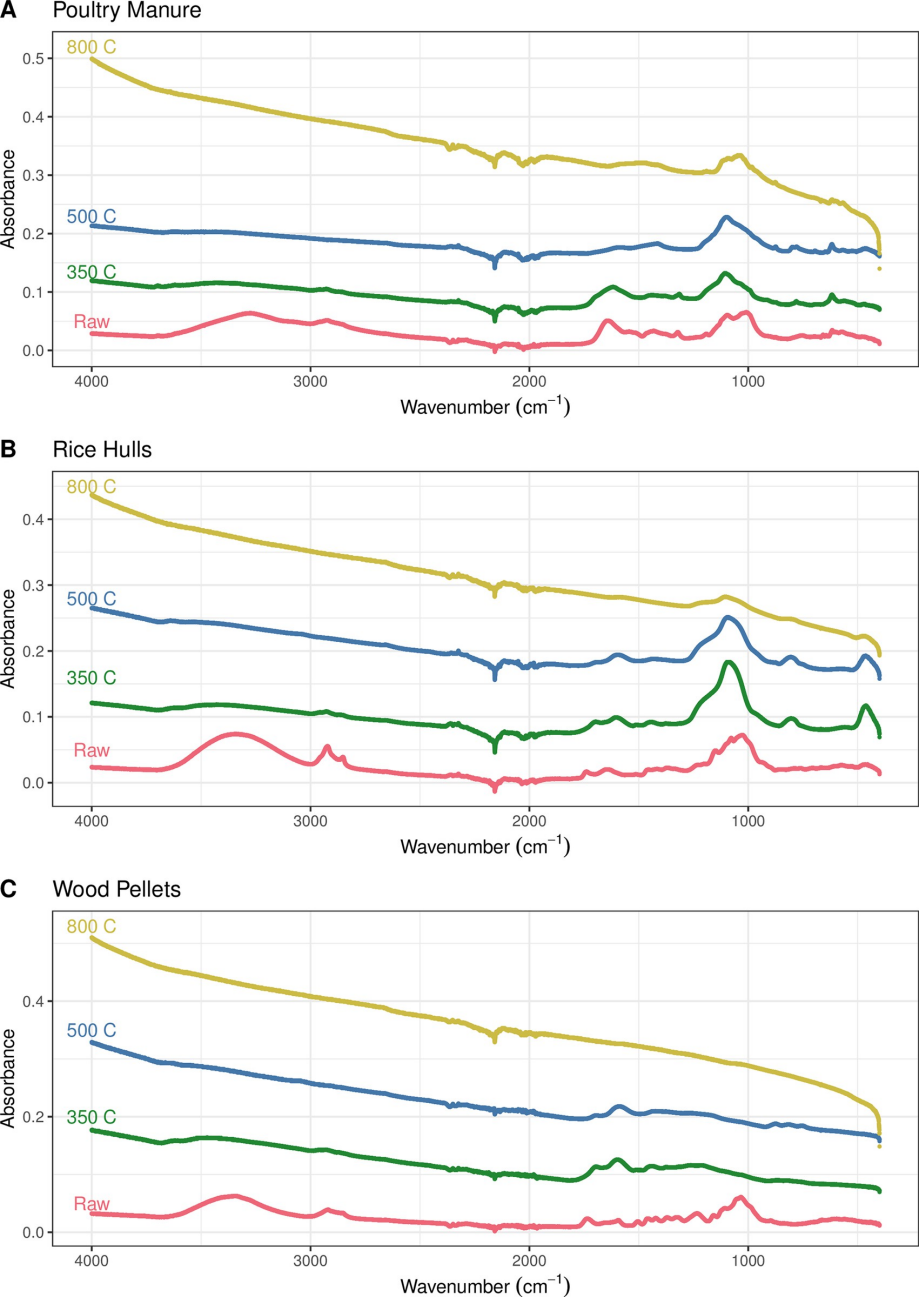
ATR-FTIR spectra for the various samples analyzed in this study, with the data grouped by feedstock (A) poultry manure, (B) rice hulls, and (C) wood pellets with the various pyrolysis treatments displayed in different colors.

Among these feedstocks, poultry manure gave higher ash contents (24%) and ash contents increased with higher pyrolysis temperatures which could result in higher pH (Table 2) [72]. A more alkaline biochar could be a result of carbonate species and/or and base cations [73], while corresponding reducing acidic functional groups like -COOH on biochar [74] (Fig 1). Such decreases in carboxylic acid groups, correlated to the increase in ash contents, has already been reported for both; manure-based biochars on pyrolysis temperature increasing from 300 to 500°C [42] as well as plant residues-based biochars, including soybean, corn, canola and peanut straw when subjected to pyrolysis at 700°C, compared to biochars at 300°C [73].

The C:N ratio was higher for both plant sources than poultry manure, highest (839) with wood pellets and increase in pyrolysis temperature decreased N contents with poultry manure, showing little variation until rapidly increasing at the highest pyrolysis temperature. The H:C ratio decreased with increasing temperature, which corresponds to increasing carbon-carbon bonding and decreasing surface moieties [75]. According to Cely et al. [76], biochar that is produced at temperatures above 400°C typically have a H:C ratio that is less than 0.5 and will further decrease with increasing pyrolysis temperatures. This was observed here with pyrolysis temperatures above 500°C having a H:C ratio less than 0.5 for all three feedstocks (Table 3). There was a strong relationship between H:C and O:C ratios (R = 0.952, P<0.01). These ratios suggest that the resulting carbon structures are being dominated by carbon-carbon bonds without extensive surface functional groups as pyrolysis temperature increases. This was also confirmed in the FTIR data, where it is clearly seen that the carbon-oxygen and carbon-hydrogen bonds become less abundant as the pyrolysis temperature is increased across all the feedstocks (Fig 1). As seen in the FTIR spectra for the 800°C pyrolysis biochars, there are no peaks corresponding to the aromatic carbon double bonds (1,580 cm^-1^) or a band due to the stretching vibrations of aromatic carbon (3,045 cm^-1^) [77,78]. These observations coupled with the low H:C ratio suggests there is no hydrogen atoms that are bonded to the carbon atoms within the biochar, thereby suggesting a structure with predominantly covalent C-C bonds (e.g., graphite) [78]. The elevated O:C ratios of lower temperature biochars suggest the presence of hydroxyl, carbonyl and carboxylate groups, which could contribute to greater CEC values [79] as CEC values are correlated with the O:C ratios [20]. The O:C ratio decreased drastically over temperature increments also observed in the FTIR data (Fig 1) through the loss of bands for oxygen surface functional groups [20,28]. There was a correlation between O:C and VM content (R = 0.907; P<0.01) and a negative correlation between O:C and fixed carbon (R = -0.811, P<0.05), which also support these conclusions. The PM and RH biochars did possess a peak near 1,100 cm^-1^, which was present in all the samples to varying degrees, associated with both the P-O bond in phosphate [80] and/or the Si-O bond [81]. This peak has been observed in other PM [82] and RH [83] biochar characterization studies. Additionally, SEM micrographs are presented in the Supplemental Information (S1-S3 Figs in S1 Text).

**Table 3:** Sorption coefficients (Linear, Langmuir, Freundlich, Dubinin–Radushkevich, and Temkin analyses) for MCPA sorption to studied biochars.

**A. Linear Isotherm**						
**Biochar**	**Linear Sorption Model** **(Henry Isotherm Analysis)**					
	**K**_**D**_ **(mL g** ^**-1**^ **)**	**SE** **(mL g** ^**-1**^ **)**	**AIC**	**RMSE**	**R** ^**2**^	**P**
**PM**_**Raw**_	3.04	0.14	131.17	7.83	0.967	<0.001
**PM350**	2.82	0.15	133.45	8.34	0.957	<0.001
**PM500**	7.74	0.41	160.94	17.90	0.954	<0.001
**PM800**						
**RH**_**Raw**_	3.73	0.14	130.72	7.7	0.976	<0.001
**RH350**	3.01	0.15	133.30	8.3	0.962	<0.001
**RH500**	5.99	0.32	155.44	15.4	0.952	<0.001
**RH800**	15.66	1.43	193.29	43.97	0.875	<0.001
**WP**_**Raw**_	10.56	0.83	180.7	31.02	0.905	<0.001
**WP350**	2.96	0.12	125.89	6.75	0.973	<0.001
**WP500**	2.45	0.21	147.14	12.20	0.890	<0.001
**WP800**	5.13	0.50	173.01	25.04	0.859	<0.001
**Notes:** SE–standard error, RMSE—root mean square error, MAE—mean absolute error, AIC—Akaike Information Criterion, BIC—Bayesian Information Criterion, P is the P-value and the corresponding Pearson’s correlation (R^2^) of the isotherm model fits. Blank values indicate that no model fit was possible of the sorption data.						

**B. Langmuir Isotherm**							
**Biochar**	**Langmuir Sorption Model** **(using Langmuiranalysis function)**						
	**Q**_**m**_ **(μg g** ^**-1**^ **)**	**K**_**L**_ **(μg g** ^**-1**^ **)**	**AIC**	**BIC**	**MAE**	**RMSE**	**R** ^**2**^
**PM**_**Raw**_	423.90	0.01	36.77	36.15	2.21	3.14	1.00
**PM350**	228.70	0.03	40.00	39.37	3.05	4.11	0.99
**PM500**	504.18	0.03	50.28	49.66	8.28	9.69	0.99
**PM800**							
**RH**_**Raw**_	3343.00	0.00	40.54	39.92	3.35	4.30	0.99
**RH350**	246.40	0.02	34.65	34.03	2.01	2.64	1.00
**RH500**	321.64	0.05	48.14	47.51	6.96	8.11	0.99
**RH800**	417.19	0.23	57.18	56.56	14.97	17.22	0.99
**WP**_**Raw**_	9501.00	0.00	53.70	53.07	9.41	12.88	0.99
**WP350**	309.20	0.02	43.86	43.23	4.67	5.67	0.98
**WP500**	156.12	0.05	54.24	53.61	11.35	13.48	0.89
**WP800**	193.54	0.28	56.81	56.19	12.85	16.70	0.94
**Notes:** SE–standard error, RMSE—relative mean square error, MAE—mean absolute error (μg g^-1^), AIC—Akaike Information Criterion, BIC—Bayesian Information Criterion, and the corresponding Pearson’s correlation (R^2^) of the isotherm model fits. Blank values indicate that no model fit was possible of the sorption data.						

**C. Freundlich Isotherm**								
**Biochar**	**Freundlich Sorption Model** **(using Freundlichanalysis function)**							
	**K**_**F**_ **(mL μg** ^**-1**^ **)**	**n**	$\frac{1}{n}$	**AIC**	**BIC**	**MAE**	**RMSE**	**R** ^**2**^
**PM**_**Raw**_	6.17	1.23	0.81	32.71	32.08	1.80	2.24	1.00
**PM350**	9.31	1.46	0.68	35.29	34.67	2.32	2.78	0.99
**PM500**	24.20	1.48	0.68	40.82	40.20	3.41	4.41	1.00
**PM800**								
**RH**_**Raw**_	4.80	1.07	0.94	39.58	38.95	3.09	3.97	0.99
**RH350**	9.48	1.44	0.70	30.67	30.05	1.53	1.89	1.00
**RH500**	26.42	1.70	0.59	31.76	31.13	1.95	2.07	1.00
**RH800**	93.50	2.26	0.44	44.91	44.28	5.58	6.19	1.00
**WP**_**Raw**_	6.61	0.86	1.16	50.19	49.57	6.72	9.62	0.99
**WP350**	8.24	1.37	0.73	39.06	38.43	2.87	3.80	0.99
**WP500**	14.50	1.87	0.54	50.93	50.31	7.66	10.23	0.91
**WP800**	49.37	2.60	0.39	49.83	49.20	7.72	9.33	0.98
**Notes:** SE–standard error, RMSE—root mean square error, MAE—mean absolute error (μg g^-1^), AIC—Akaike Information Criterion, BIC—Bayesian Information Criterion, P–P-value, and the corresponding Pearson’s correlation (R^2^) of the isotherm model fits. Blank values indicate that no model fit was possible of the sorption data.						

**D. Dubinin-Radushkevich Isotherm**								
**Biochar**		**Dubinin–Radushkevich Sorption Model** **(using dubininradushkevichvanalysis function)**						
	**Q**_**DR**_ **(μg g** ^**-1**^ **)**	**K**_**DR**_ **(mol**^**2**^ **J**^**-2**^**)**	**E** **(J mol** ^**-1**^ **)**	**AIC**	**BIC**	**MAE**	**RMSE**	**R** ^**2**^
**PM**_**Raw**_	110.30	1.23E-05	201.6	55.29	54.67	11.71	14.71	0.90
**PM350**	101.20	7.51E-06	258.0	54.37	53.75	10.19	13.63	0.90
**PM500**	211.80	4.45E-06	335.2	64.24	63.61	24.89	31.01	0.89
**PM800**								
**RH**_**Raw**_	191.30	6.50E-05	87.7	55.96	55.33	9.27	15.55	0.95
**RH350**	108.30	8.12E-06	248.1	53.89	53.26	10.09	13.09	0.92
**RH500**	179.90	3.82E-06	361.8	61.67	61.05	19.70	25.04	0.90
**RH800**								
**WP**_**Raw**_	421.70	4.12E-05	110.2	61.93	61.30	14.50	25.58	0.97
**WP350**	103.90	8.43E-06	243.5	56.40	55.78	12.54	16.14	0.87
**WP500**	82.86	2.16E-06	481.1	58.30	57.67	15.25	18.90	0.76
**WP800**	156.60	5.09E-07	991.1	61.79	61.17	21.62	25.29	0.87
**Notes:** SE–standard error, RMSE—relative mean square error, MAE—mean absolute error (μg g^-1^), AIC—Akaike Information Criterion, BIC—Bayesian Information Criterion, P–P-value, and the corresponding Pearson’s correlation (R^2^) of the isotherm model fits. Blank values indicate that no model fit was possible of the sorption data.						

**E. Temkin Isotherm**								
**Biochar**		**Temkin Isotherm Analysis** **(using temkinanalysis function)**						
	**A**_**t**_ **(L mg** ^**-1**^ **)**	**B**_**t**_ **(J mol** ^**-1**^ **)**	**AIC**	**BIC**	**MAE**	**RMSE**	**R** ^**2**^
**PM**_**Raw**_	3.58	146.01	61.79	60.80	21.83	24.53	0.69
**PM350**	4.34	152.13	61.79	58.87	18.67	20.89	0.74
**PM500**	10.91	84.63	61.79	68.42	41.09	46.28	0.71
**PM800**							
**RH**_**Raw**_	3.75	131.88	61.79	63.30	27.04	30.22	0.64
**RH350**	4.41	145.23	61.79	59.61	19.35	22.22	0.74
**RH500**	11.84	98.21	61.79	65.35	31.60	35.83	0.76
**RH800**							
**WP**_**Raw**_	4.59	67.48	61.79	72.83	58.61	66.84	0.58
**WP350**	4.56	151.12	61.79	59.60	19.90	22.19	0.72
**WP500**	14.75	200.07	61.79	58.10	17.33	19.58	0.73
**WP800**	66.30	126.01	61.79	62.62	25.78	28.56	0.83
**Notes:** A_t_ and B_t_ are the two Temkin coefficients, RMSE—root mean square error, MAE—mean absolute error, AIC—Akaike Information Criterion, BIC—Bayesian Information Criterion, and the corresponding Pearson’s correlation (R^2^) of the isotherm model fits. Blank values indicate that no model fit was possible of the sorption data.						

These results confirm the findings of previous studies which described the dependence of biochar properties on pyrolysis temperature and feedstock [e.g., 84]. These variations in response from different feedstocks, after been subjected to various temperatures, could lead to different outcomes with pesticide sorption. While, on one hand, high pyrolysis temperature could provide more pesticide sorption due to enhanced surface area; on the other hand, reduced functional groups on the biochar surface due to high pyrolysis temperatures could lower pesticide sorption. This was observed in this study by the decreases observed in the estimates from the Langmuir isotherm for the maximum sorbed quantity (Q_m_) for MCPA, which had decreases for the biochars for WP and RH feedstocks, regardless of the pyrolysis temperature. The study of these factors on pesticide sorption is therefore necessary to determine a biochar with desirable characteristics for pesticide removal [28].

### Sorption results

**Fig 2:**
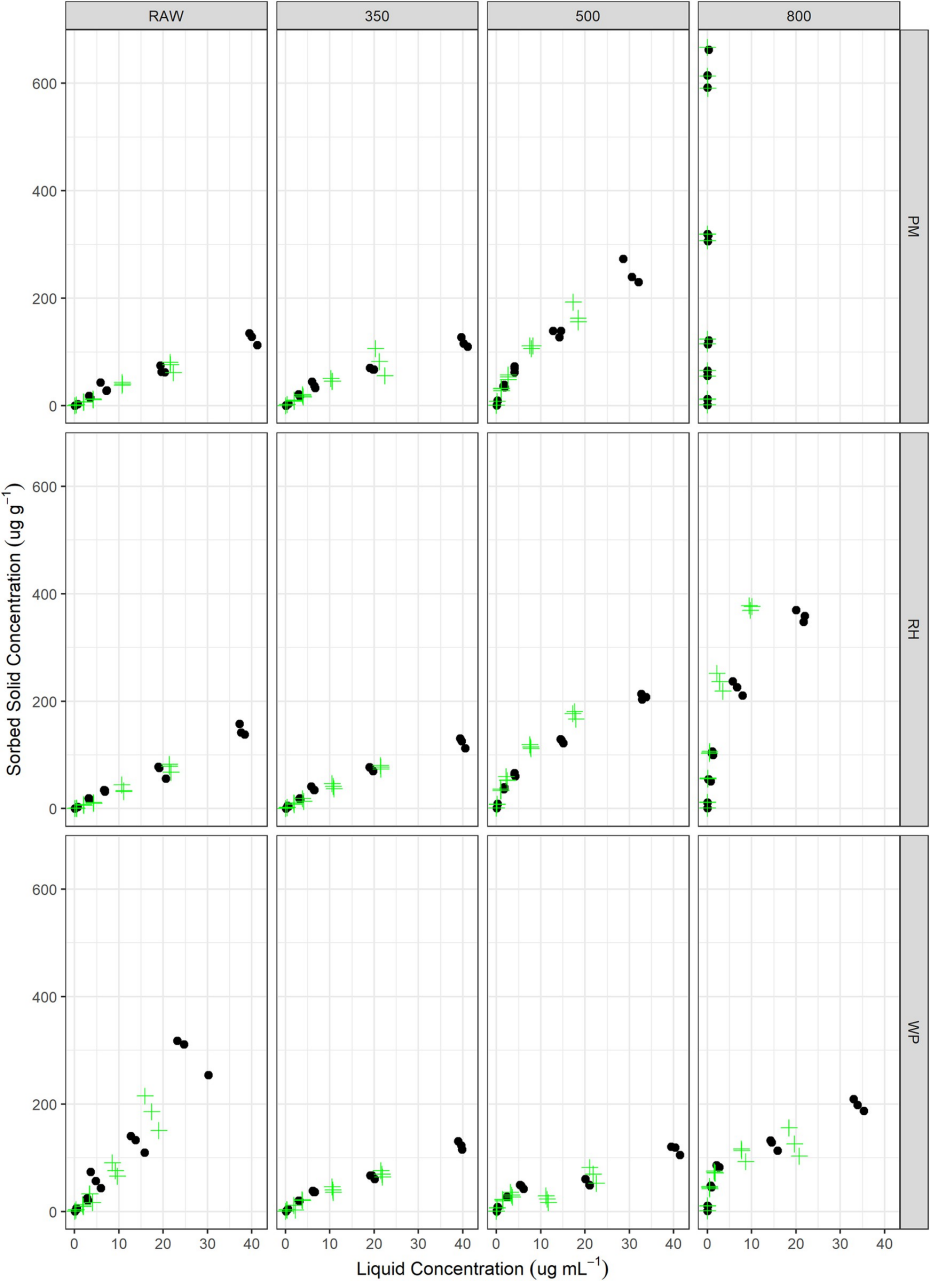
Data for individual sorption and desorption experiments for the three feedstock materials [poultry manure (PM), rice hulls (RH), and wood pellets (WP)] as well as the various pyrolysis temperatures (Raw, 350, 500, and 800°C). The sorption data points are shown with a black dot and the desorption data points are illustrated with the green plus signs.

The raw data are shown in Fig 2, while the estimated parameters for the five sorption models are summarized in Table 3. Examining the resulting linear sorption coefficients (K_D_), both feedstock and temperature had a statistically significant effect on the resulting sorption of MCPA (S3 Table in S1 Text; Supplemental Information). K_D_ for MCPA increased tremendously with increasing pyrolysis temperature from 350 to 800°C. However, only the 800°C was statistically significant (P<0.05; S4 Table in S1 Text).

The resulting individual isotherm model analyses are presented in the Supplemental Information (S4-S16 Figs in S1 Text). Based on the assessment of the AIC criterion [85,86], along with the BIC, MAE, RMSE, and R^2^ (Table 3), the Freundlich isotherm model was the best fit describing the nature of MCPA sorption to the various biochars and feedstocks. This would infer that the sorption sites are dissimilatory in their sorption energy [87]. In general, lower pyrolysis temperature typically reduced the estimated sorption capacity (lower K_D_ and Q_m_ values), although not statistically significant across all the feedstocks. Except for the PM feedstock, the value of $\frac{1}{n}$ typically decreased with increasing pyrolysis temperatures, indicating a more non-linear behavior of the resulting isotherm and greater heterogeneity of sorption sites (Fig 2). However, direct comparison of the Freundlich coefficients is hampered by differing values of n across the different sorbents [88].

The greater increase in the sorption coefficients of the PM and RH 800°C biochars could be related to the interaction with mineral phases (chemosorption) versus the aromatic carbon orbitals, due to the lack of similar sorption increases with the WP800 biochar (Table 3). Additionally, there is no statistically significant correlation between sorption coefficients and the surface area of the biochar. This would be in agreement with the importance of clay mineral sorption that has been observed in soil matrices [89] and lack of direct correlation of MCPA sorption and soil organic matter (carbon) content [52]. However, no statistically significant correlation was observed between any of the sorption coefficients and the ash content of the various biochars (S3 Table in S1 Text). MCPA has a pK_a_ of 3.1 (Table 1) and therefore at the pH of the biochar solutions used here (Table 2; pH>6) MCPA would be in the anionic form, which could provide a mechanism for cation interactions [90]. It is also interesting to note that the maximum Langmuir’s sorption capacity (Q_m_) for MCPA is the highest for the raw feedstocks for the RH and WP, and within the same order of magnitude for the PM treatments (Table 3B). Similarly, the K_D_ estimated from the linear relationship between Q_s_ and Q_e_ (Table 3A) also suggests minimal increases as a function of pyrolysis temperature, except for the PM800 biochar (Table 3A). There was a high sorption capacity observed for the raw WP feedstock (9,501 ug g^-1^; Table 3B), although the exact mechanisms resulting in this elevated sorption capacity were not elucidated. Overall, the PM800 biochar possessed the highest sorption capacity and removed nearly 100% of the MCPA at all concentrations evaluated here (Fig 2). There was an increasing trend in the estimated free sorption energy from the Dubinin–Radushkevich model (Table 3D) with pyrolysis temperatures. However, we cannot confirm this across all feedstocks due to the lack of fit of the Dubinin–Radushkevich isotherm for PM800 and RH800. This increasing trend in energy would suggest a stronger attraction of MCPA to higher pyrolysis temperatures, but still these values are below those typical for chemosorption (>20 kJ mol^-1^) [91].

There was a negative correlation with O and VM content and a corresponding positive correlation with H content and H:C ratios with the Langmuir sorption coefficient as well as the Freundlich coefficients, which negates the importance of oxygen surface groups in the sorption mechanisms and highlights potential competitive sorption with VM material already sorbed to the biochars (S17 Fig in S1 Text). The values for the K_D_ for the feedstocks and biochars (2.8 to 921 L kg^-1^; Table 3) are greater than those typically observed for MCPA sorption to soils (0.3 to 1.5 L kg^-1^) [52–55].

Rice residues-based biochars have already been reported to increase sorption capacity for pesticides [26,92–94]. Ren et al. [95] also found better sorption capacity of rice biochar for carbaryl with increasing pyrolysis temperature, with increased sorption obtained at 700°C. Manna and Singh [94] also reported more pyrazosulfuron-ethyl sorption on higher pyrolysis temperatures. In comparison to 5.3–8.6% pesticide sorption with unamended soil, they recorded 7.5–50.4% and 55.9–91.8% sorption, when amended with biochars pyrolyzed at 400°C and 600°C, respectively. Wang et al. [96] had also found higher sorption capability for terbuthylazine by pine wood biochar pyrolyzed at 700°C, compared with biochar produced at 350°C, which they linked to higher porosity and surface area for the higher temperature biochar. A wheat straw biochar at 300°C was observed to have a K_f_ for MCPA of 43.7 mg^1-n^kg^-1^L^n^ [11] and a switchgrass 425°C biochar was observed to have a K_f_ for MCPA of 2.3 mg^1-n^kg^-1^L^n^ [97], which are comparable to those observed in our study (Table 3).

### Desorption results

The desorption Freundlich coefficients are shown in Table 4. There was a strong correlation between the sorption coefficients of the sorption models evaluated (K_D_, K_L,_ K_DR_, A, and K_f_) and the desorption K_f_ (Table 4). Results showed that sorbed MCPA on studied biochars was variably desorbed back to solution depending on the feedstock as well as the concentration level of the herbicide (S5 Table in S1 Text). Among all the twelve testing adsorbers, raw feedstocks possessed weakest sorption of MCPA because a large proportion of initially sorbed herbicide on material surface desorbed back during first desorption cycle (S5 Table in S1 Text), as well as the raw feedstocks possessing the lowest free energy of sorption (E; Table 3D). The reversible sorption from raw feedstock surfaces and from biochars pyrolyzed at low temperatures might be related to the presence of micropores, serving as entrapping sites for pesticide molecules [98,99]. However, MCPA desorption became more irreversible with increases in pyrolysis temperature, as supported by the decreasing values of H (Table 4) and increasing values of E (Table 3). All three feedstocks subjected to the higher temperature of 800°C desorbed back only minute amounts, especially the poultry manure and rice hull based biochars, which produced negative desorption percentages (S5 Table in S1 Text). This demonstrates not only absence of any reversible MCPA sorption, but continued sorption was evident for PM800 and RH800 despite the reduced solution concentrations. All values of H were close to the value of 1, except for the 800°C biochars, which possessed lower H values (Table 4), which suggests some degree of irreversibility in the sorption-desorption of MCPA on the highest temperature biochars. This lack of desorption would be consistent with more chemical-like interactions (i.e., chemosorption) versus physical and/or electrostatic mechanisms. Although this cannot be confirmed due to the lack of isotherm fits (Temkin and Dubinin–Radushkevich) for the PM800 and RH800 samples. Higher desorption of MCPA from raw feedstocks and those of biochar pyrolyzed at low temperatures, particularly rice hulls and wood pellets, could be ascribed to lower energy of sorption resulting from physical and/or electrostatic interactions, reduced surface areas found in these materials [100], or differences in the pH values of the solutions (Table 2).

**Table 4:** Freundlich model coefficients for MCPA desorption from studied biochars and feedstocks.

Biochar	Freundlich Desorption Model (Freundlich Analysis)								
	K_F_ (mL μg^-1^)	$\frac{1}{n}$	AIC	BIC	MAE	RMSE	R^2^	H
**PM**_**Raw**_	3.84	0.96	30.82	30.20	1.33	1.91	0.99	1.18
**PM350**	6.04	0.85	25.48	24.86	1.02	1.23	1.00	1.25
**PM500**	30.48	0.60	33.93	33.31	2.17	2.48	1.00	0.88
**PM800**								
**RH**_**Raw**_	2.48	1.12	22.67	22.05	0.68	0.97	1.00	1.19
**RH350**	5.00	0.89	-3.38	-4.01	0.09	0.11	1.00	1.28
**RH500**	34.77	0.57	34.92	34.30	2.27	2.69	1.00	0.96
**RH800**	143.20	0.43	50.77	50.14	8.26	10.09	1.00	0.97
**WP**_**Raw**_	4.70	1.28	30.36	29.73	1.45	1.84	1.00	1.10
**WP350**	6.72	0.76	27.21	26.59	0.95	1.42	1.00	1.04
**WP500**	13.63	0.47	50.43	49.80	7.32	9.81	0.80	0.87
**WP800**	56.20	0.29	45.36	44.73	5.21	6.43	0.98	0.75

**Notes:** SE–standard error, RMSE—root mean square error, MAE—mean absolute error(μg g^-1^), AIC—Akaike Information Criterion, BIC—Bayesian Information Criterion, P–P-value, and the corresponding Pearson’s correlation (R^2^) of the isotherm model fits. Blank values indicate that no model fit was possible of the sorption data.

## Conclusion

MCPA sorption differs among feedstocks and biochars behave differently for MCPA sorption depending on the pyrolysis temperature. In general, higher pyrolysis temperatures did increase the sorption capacity. However, all three raw feedstocks used in this study did have good potential of MCPA sorption (K_D_ ranging from 3 to 11 mL g^-1^; Table 3). Raw wood pellets possessed the highest K_D_ and Q_m_ of all materials tested here, which also possessed the largest proportion of reversible sorption. This fact would suggest that for MCPA sorption in the field setting, raw WP feedstock would act superior as compared to the biochars created here due to the largest sorption capacity and the reversible nature of the sorption (H = 1.10). This would also reduce overall costs, although the durability of these amendments would be limited due to microbial mineralization. Poultry manure biochar could be regarded as one with superior MCPA sorption properties when subjected to higher pyrolysis temperatures (800°C). This treatment removed nearly 100% of all the MCPA from solution for the concentrations evaluated and was irreversible sorbed. However, due to the lack of this behavior in the WP800 the removal is hypothesized to be due to the mineral phases (cation bridging) and not the carbon-backbone of the biochar (π-π orbital interactions). This type of biochar (PM800) could be useful when sudden losses of herbicides need to be addressed (e.g., point source leaks, spills) and would increase the economic feasibility of using higher pyrolysis temperatures for this type of remediation versus whole field applications. Pyrolysis temperature should be reduced below 500°C when biochars are needed to sorb MCPA to reduce field runoff losses associated with heavy irrigation or rainfall. Desorption of herbicides with time will aid in maintaining efficacy for weed control such as those observed in the raw feedstocks or the PM350 or RH350 biochars.

## Supporting information

S1 Text A Microsoft word document containing the supplemental figures and tables as summarized below. **S1-S3 Figs** Scanning electron micrographs of all the various biochar and feedstock samples used in thus experiment. **S4 Fig** Linear fit of all sorption data. **S5-S7 Figs** Non-linear curve fits the Langmuir sorption models for the 3 different feedstocks. **S8-S10 Figs** Non-linear curve fits the Freundlich sorption models for the 3 different feedstocks. **S11-S13 Figs** Non-linear curve fits the Temkin sorption models for the 3 different feedstocks. **S14-S16 Figs** Non-linear curve fits of the Dubinin–Radushkevich sorption models for the 3 different feedstocks. **S17 Fig**–Correlation matrix for the measured variables. **S1 Table.** Details of liquid additions for each concentration level evaluated. **S2 Table:** Description of sorption model equations and linearized forms used within the PUPAIM package. **S3 Table.** Analysis of variance for the dependence of the feedstock and pyrolysis temperature on the resulted observed values of K_D_ for the entire experiment. **S4 Table.** Percentage of MCPA desorption from studied biochars. **S5 Table:** ANOVA analysis for sorption coefficient (K_D_) and the influence of feedstock and pyrolysis temperature.
(DOCX)

S2 Text An example R script for generating Langmuir sorption figures in the manuscript from the raw data files in R. Props. **xlsx**–An EXCEL file with the raw data on the biochar characterization along with the fitted sorption curve coefficients. Used for determining the relationships between biochar properties and sorption coefficients. **MCPA.xlsx**—An Excel datafile with all the collected raw sorption and desorption data. **FTIR.CSV**–A CSV file with the data from the ATR_FTIR analysis.
(TXT)

S1 File (CSV)

S2 File (XLSX)

S3 File (XLSX)
