# Correction: 2-Methyl-4-chlorophenoxyacetic acid (MCPA) sorption and desorption as a function of biochar properties and pyrolysis temperature

**DOI:** 10.1371/journal.pone.0308504

**Published:** 2024-08-05

**Authors:** Abdullah Niaz, Kurt A. Spokas, Beatriz Gámiz, David Mulla, Khaliq R. Arshad, Sarfraz Hussain

The third author’s name is spelled incorrectly. The correct name is: Beatriz Gámiz. The correct citation is: Niaz A, Spokas KA, Gámiz B, Mulla D, Arshad KR, Hussain S (2023) 2-Methyl-4-chlorophenoxyacetic acid (MCPA) sorption and desorption as a function of biochar properties and pyrolysis temperature. PLOS ONE 18(9): e0291398. https://doi.org/10.1371/journal.pone.0291398.

There are errors in the author affiliations. The correct affiliations are as follows:

Abdullah Niaz^1,2^, Kurt A. Spokas^3^, Beatriz Gámiz^4, 5^, David Mulla^2^, Khaliq R. Arshad^1^, and Sarfraz Hussain^1^

**1** Pesticide Residue Laboratory, Institute of Soil Chemistry & Environmental Sciences, Kala Shah Kaku, Punjab, Pakistan, **2** University of Minnesota, Department of Soil, Water and Climate, St. Paul, MN USA, **3** United States Department of Agriculture, Agricultural Research Service, St. Paul, MN USA, **4** Instituto de Recursos Naturales y Agrobiología de Sevilla (IRNAS), Spanish National Research Council (CSIC), Seville, Spain, **5** Department of Inorganic Chemistry, Chemical Institute for Energy and the Environment (IQUEMA), University of Córdoba, Córdoba, Spain.
