# Supplementary material for: 2-Methyl-4-chlorophenoxyacetic acid (MCPA) sorption and desorption as a function of biochar properties and pyrolysis temperature
Source: PLoS One. 2023 Sep 8;18(9):e0291398. doi: 10.1371/journal.pone.0291398 (PMC10490996; doi:10.1371/journal.pone.0291398)
Supplement: S1 Text — S1-S3 Figs Scanning electron micrographs of all the various biochar and feedstock samples used in thus experiment. S4 Fig Linear fit of all sorption data. S5-S7 Figs Non-linear curve fits the Langmuir sorption models for the 3 different feedstocks. S8-S10 Figs Non-linear curve fits the Freundlich sorption models for the 3 different feedstocks. S11-S13 Figs Non-linear curve fits the Temkin sorption models for the 3 different feedstocks. S14-S16 Figs Non-linear curve fits of the Dubinin–Radushkevich sorption models for the 3 different feedstocks. S17 Fig–Correlation matrix for the measured variables. S1 Table. Details of liquid additions for each concentration level evaluated. S2 Table: Description of sorption model equations and linearized forms used within the PUPAIM package. S3 Table. Analysis of variance for the dependence of the feedstock and pyrolysis temperature on the resulted observed values of KD for the entire experiment. S4 Table. Percentage of MCPA desorption from studied biochars. S5 Table: ANOVA analysis for sorption coefficient (KD) and the influence of feedstock and pyrolysis temperature. (DOCX) [file pone.0291398.s001.docx]

Supplemental Information for :

**2-METHYL-4-CHLOROPHENOXYACETIC ACID (MCPA) SORPTION AND DESORPTION AS A FUNCTION OF BIOCHAR PROPERTIES AND PYROLYSIS TEMPERATURE**

A. Niaz ^1,2^, K.A. Spokas ^3^, B. Gámiz ^4^, D. Mulla ^2^, , K.R. Arshad ^1^, and S. Hussain ^1^

1 - Pesticide Residue Laboratory, Institute of Soil Chemistry & Environmental Sciences,

Kala Shah Kaku, Punjab, Pakistan.

2 – University of Minnesota, Department of Soil, Water and Climate, St. Paul, MN USA

3 – United States Department of Agriculture, Agricultural Research Service, St. Paul, MN USA

4 - Instituto de Recursos Naturales y Agrobiología de Sevilla (IRNAS), Spanish National Research Council (CSIC), Seville, Spain

(A) (B)


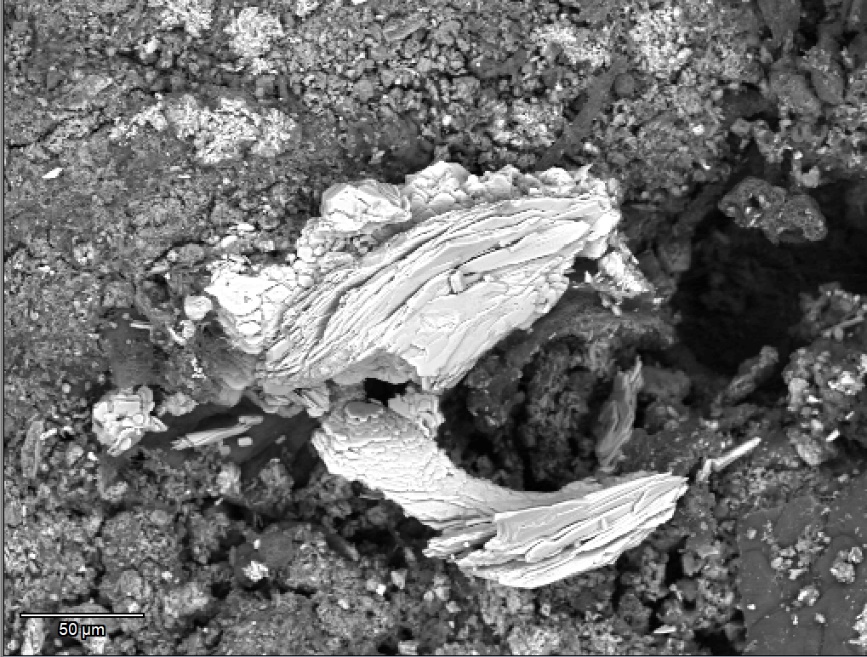

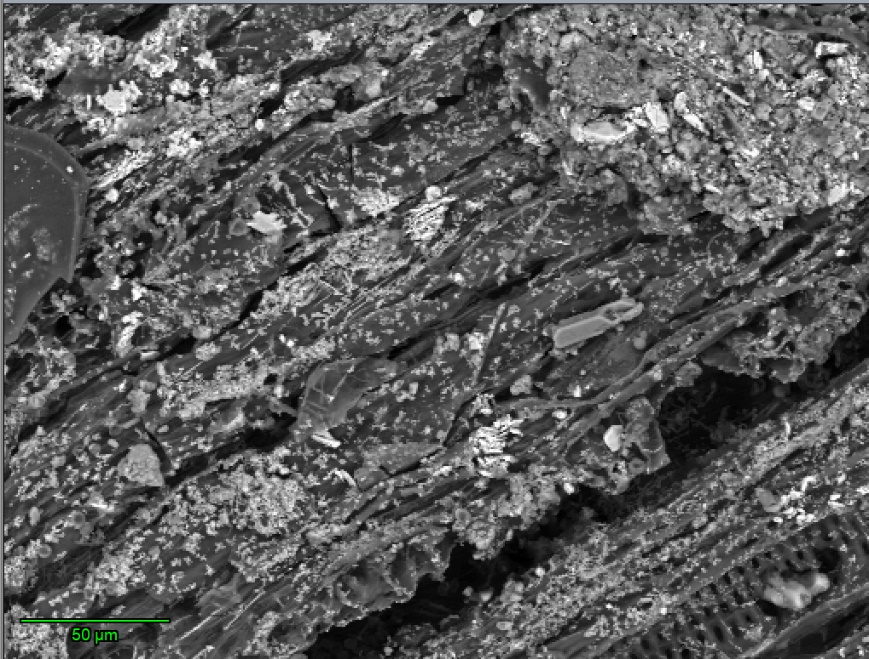


(C) (D)


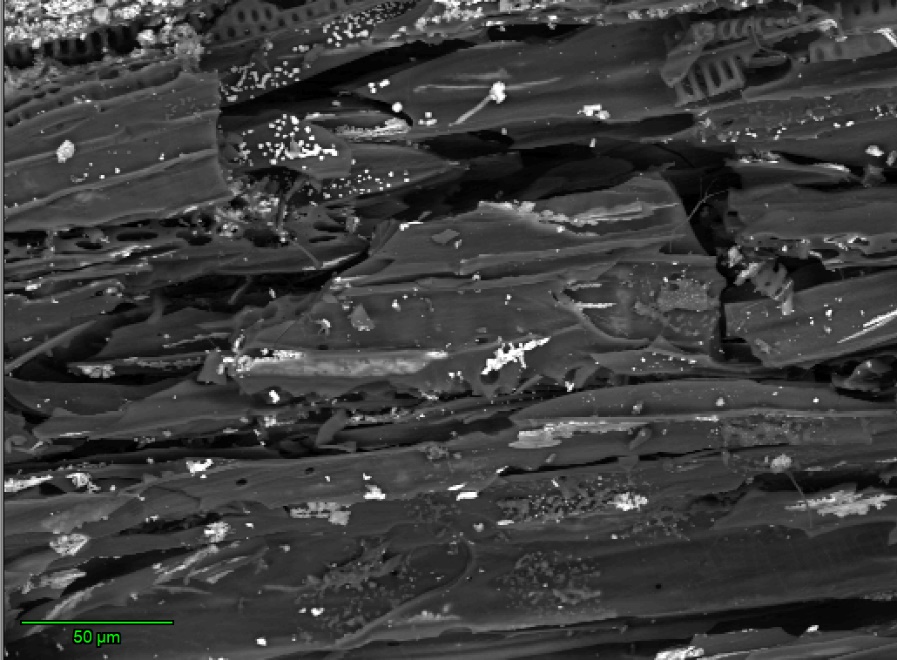

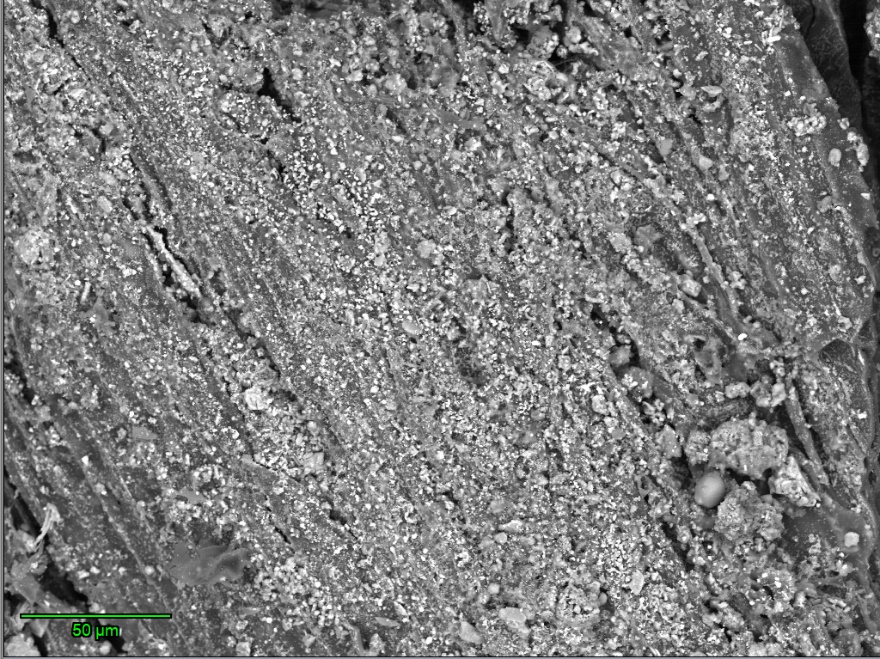


**Figure. S1 –** Scanning electron micrographs for poultry manure feedstock of (A) original feedstock, (B) 350 ^o^C biochar, (C) 500 ^o^C biochar, and (D) 800 ^o^C biochar. Scale bar is shown in the bottom left corner of the images.

(A) (B)


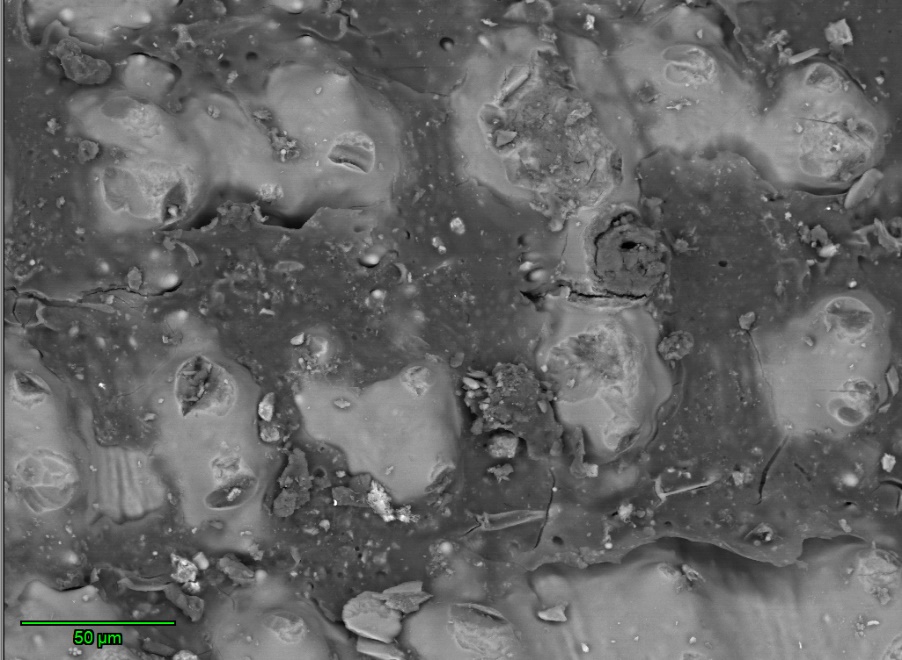

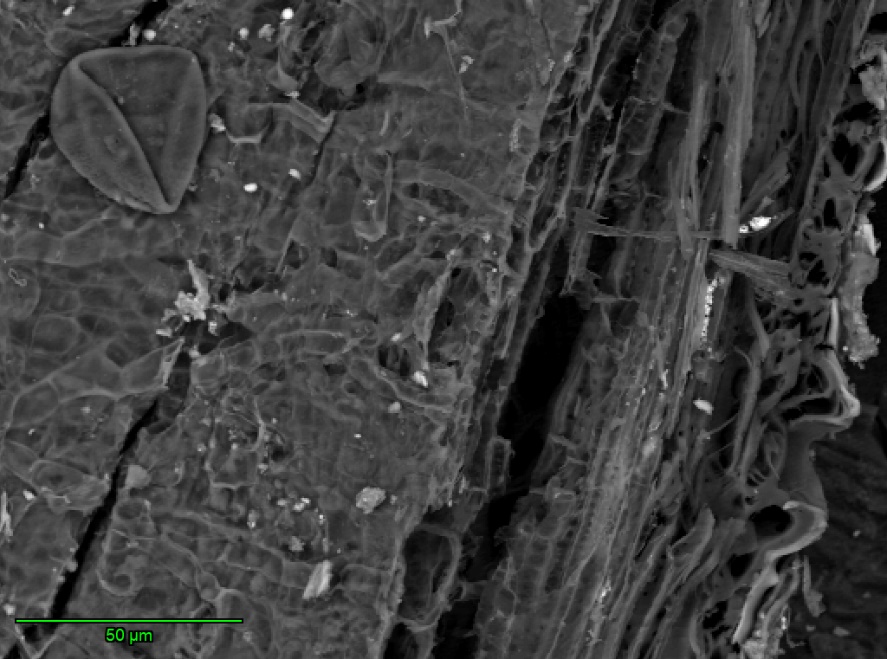


(C) (D)


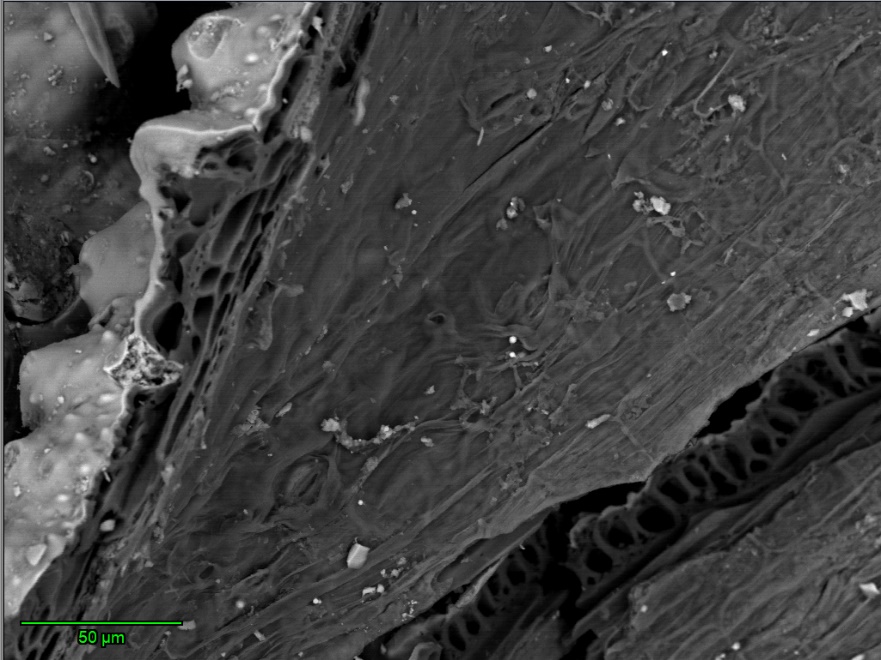

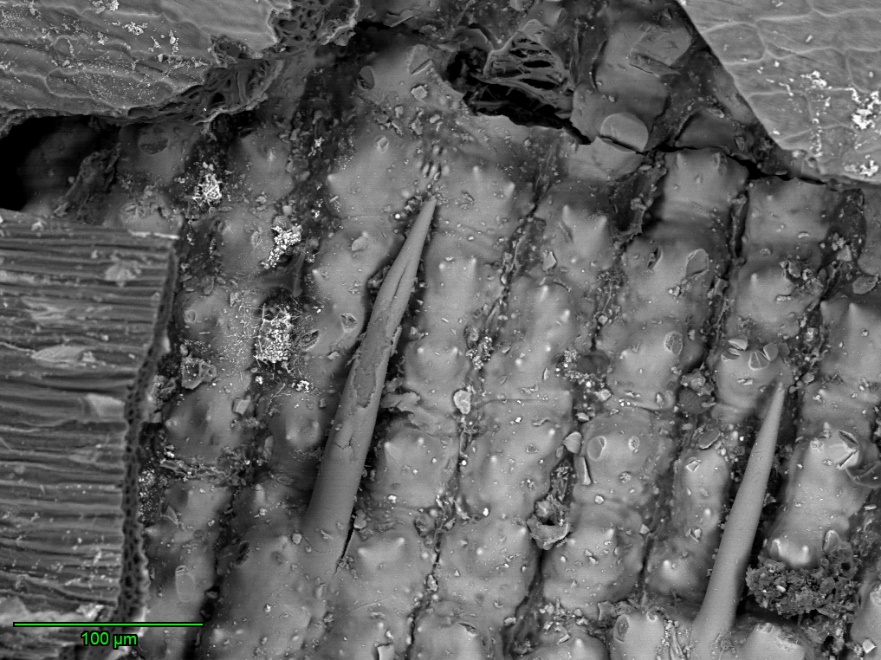


**Figure. S2 –** Scanning electron micrographs for rice hull feedstock of (A) original feedstock, (B) 350 ^o^C biochar, (C) 500 ^o^C biochar, and (D) 800 ^o^C biochar. Scale bar is shown in the bottom left corner of the images.

(A) (B)


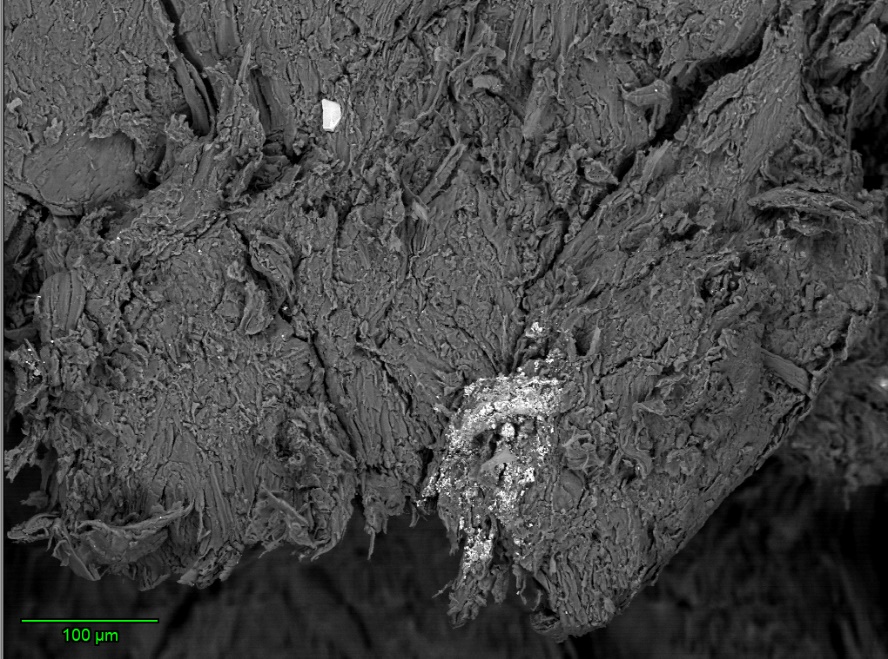

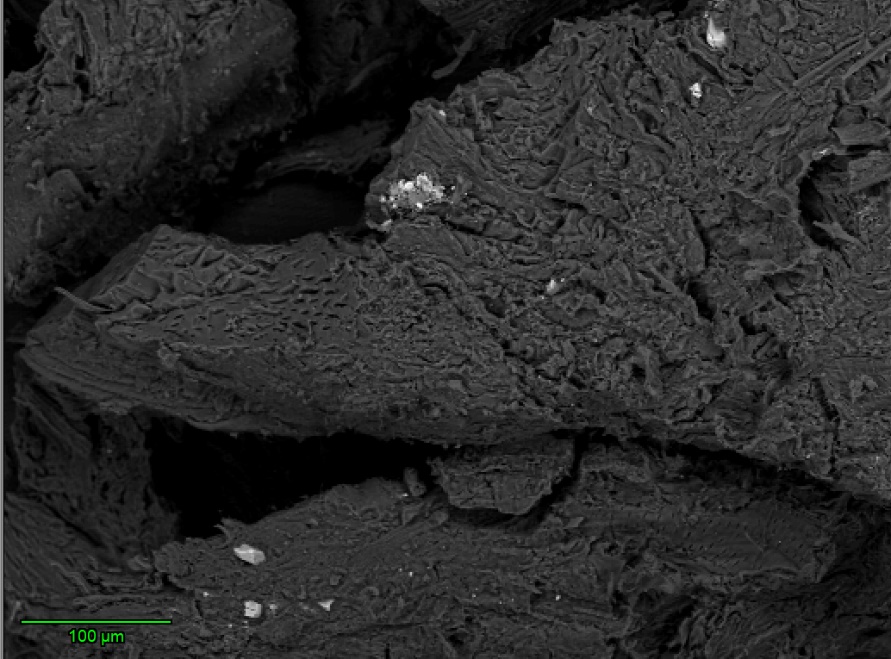


(C) (D)


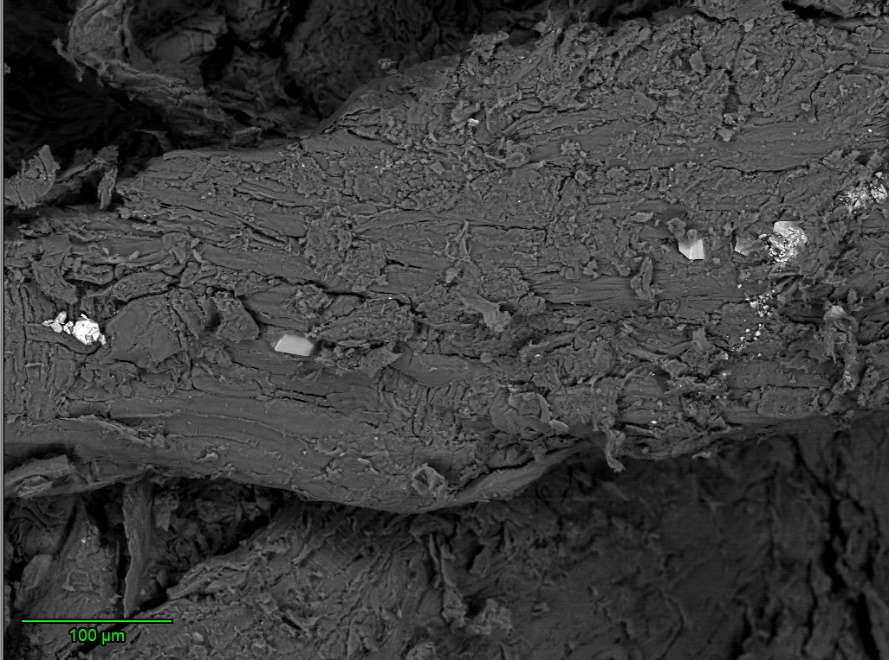

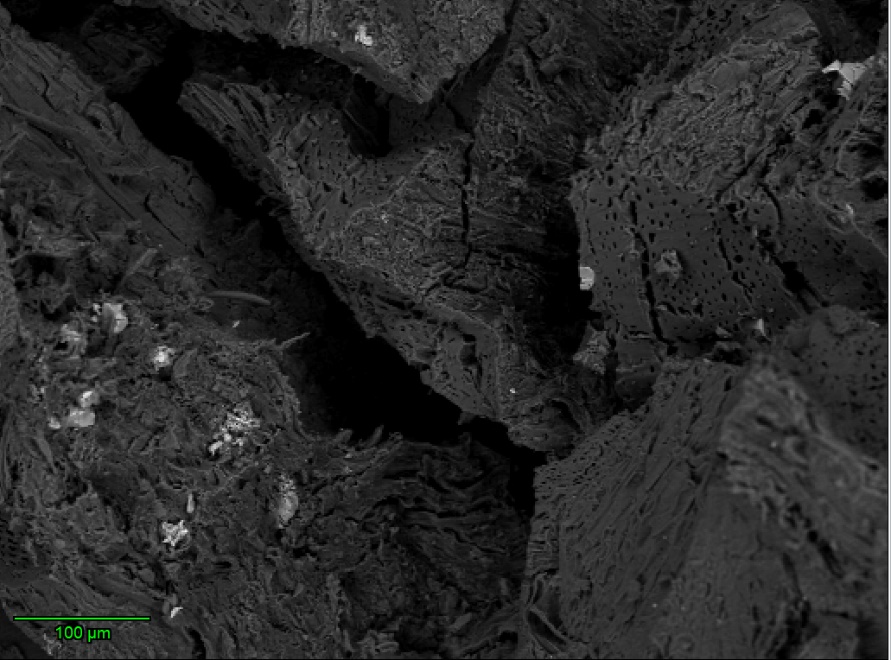


**Figure. S3 –** Scanning electron micrographs for wood pellet feedstock of (A) original feedstock, (B) 350 ^o^C biochar, (C) 500 ^o^C biochar, and (D) 800 ^o^C biochar. Scale bar is shown in the bottom left corner of the images.


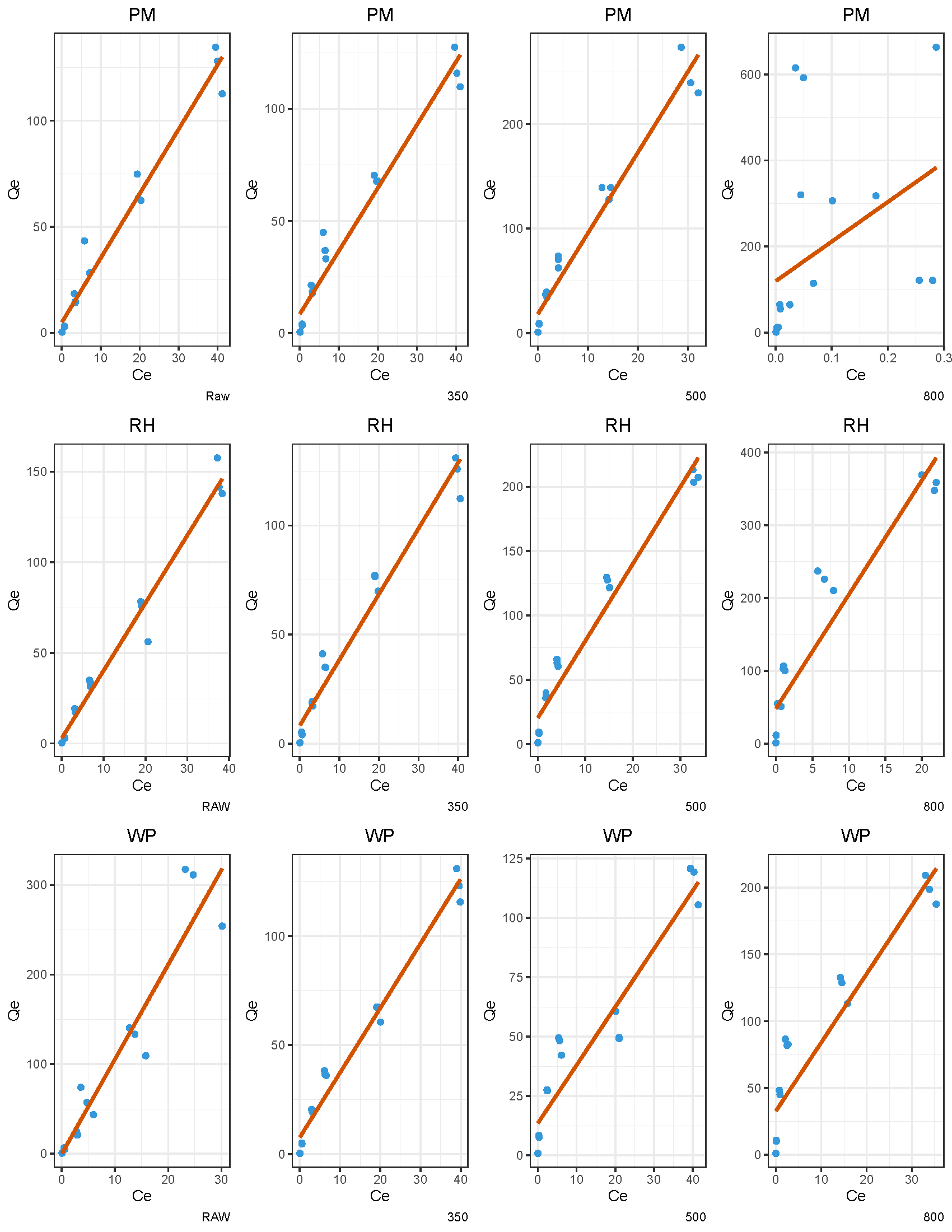


**Figure S4**. Example of linear fits of the sorbed solid concentration at equilibrium (Q_e_) and the resulting liquid concentration (C_e_) from each of the feedstock and biochar samples evaluated in this study. The feedstock type is abbreviated in the title (WP – wood pellets; RH- Rice hull-; PM – poultry manure) and the pyrolysis temperature is given in the lower right footnote to each graph. The resulting K_D_ values are given in Table 3.


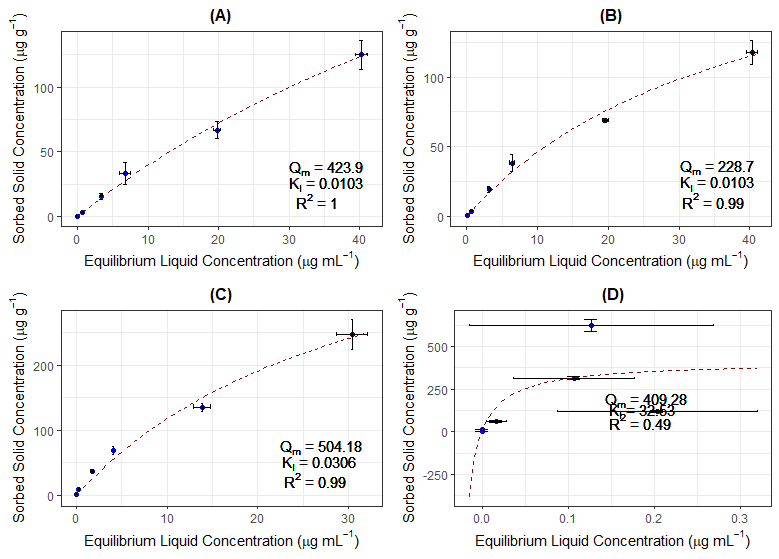


**Figure S5**. Example of non-linear fits of the Langmuir isotherm with the solid concentration at equilibrium (Q_e_) and the resulting liquid concentration (C_e_) from each of the (A) poultry litter feedstock, (B) PM350, (C) PM500, and (D) PM800 biochar samples evaluated in this study. The resulting coefficient values are presented on the figures and summary of the statistical assessments are given in Table 3.


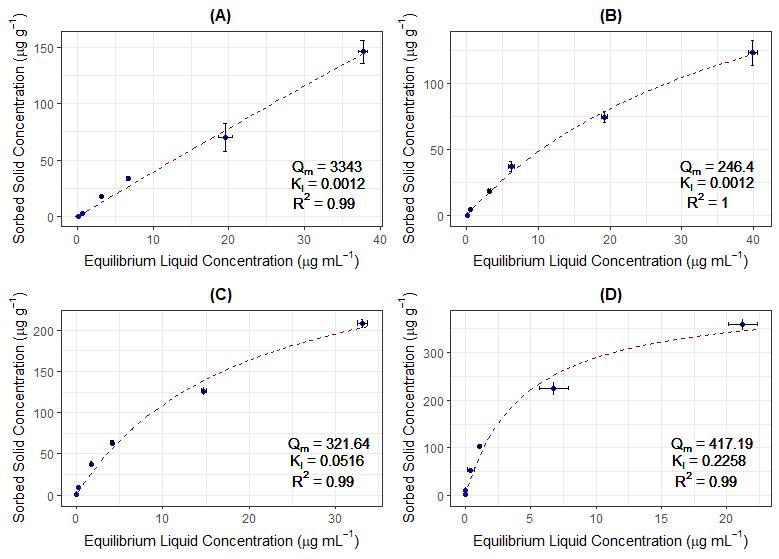


**Figure S6**. Example of non-linear fits of the Langmuir isotherm with the solid concentration at equilibrium (Q_e_) and the resulting liquid concentration (C_e_) from each of the (A) rice hull feedstock, (B) RH350, (C) RH500, and (D) RH800 biochar samples evaluated in this study. The resulting coefficient values are presented on the figures and summary of the statistical assessments are given in Table 3.


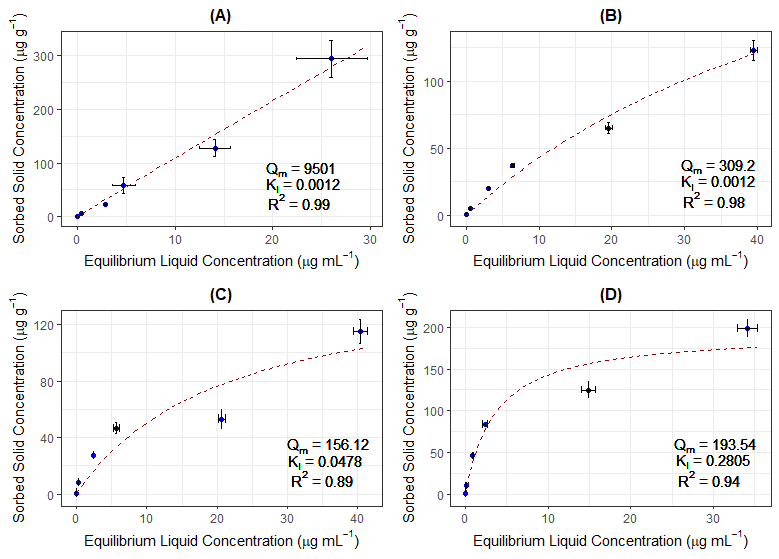


**Figure S7**. Example of non-linear fits of the Langmuir isotherm with the solid concentration at equilibrium (Q_e_) and the resulting liquid concentration (C_e_) from each of the (A) wood pellet feedstock, (B) WP350, (C) WP500, and (D) WP800 biochar samples evaluated in this study. The resulting coefficient values are presented on the figures and summary of the statistical assessments are given in Table 3.

**
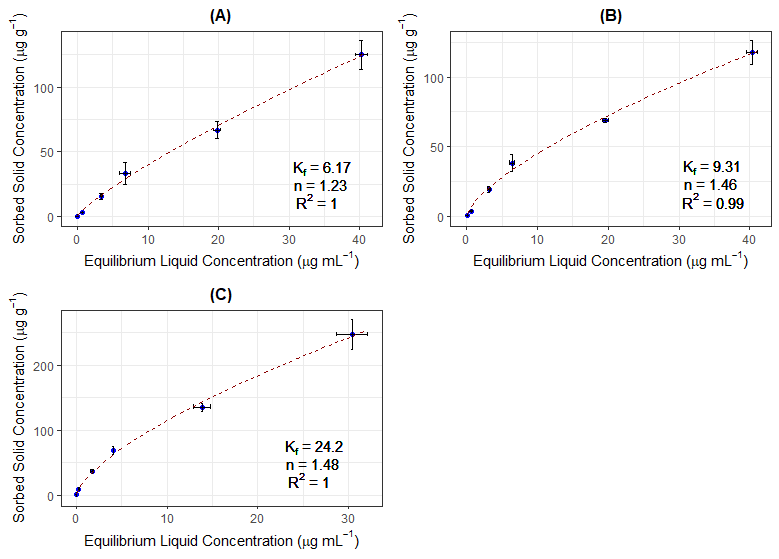
**

**Figure S8**. Example of non-linear fits of the Freundlich isotherm with the solid concentration at equilibrium (Q_e_) and the resulting liquid concentration (C_e_) from each of the (A) poultry litter feedstock, (B) PM350, and (C) PM500 evaluated in this study. The PM800 did not fit the Freundlich isotherm. The resulting coefficient values are presented on the figures and summary of the statistical assessments are given in Table 3.


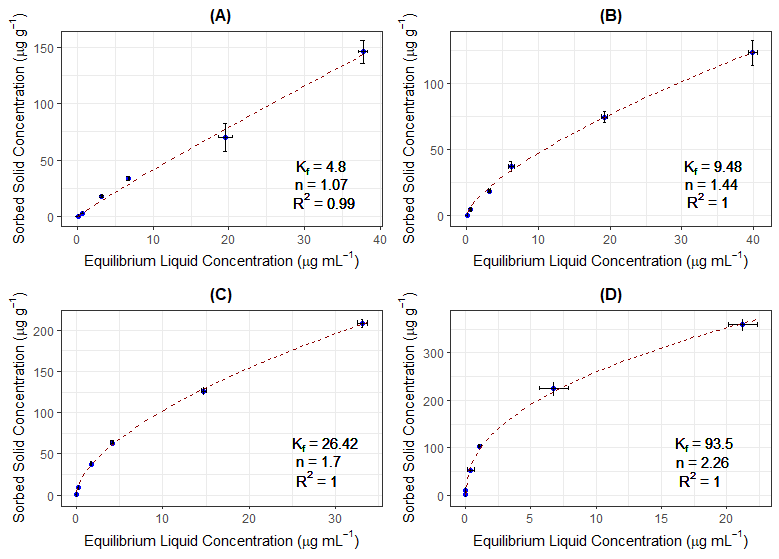


**Figure S9**. Example of non-linear fits of the Freundlich isotherm with the solid concentration at equilibrium (Q_e_) and the resulting liquid concentration (C_e_) from each of the (A) rice hull feedstock, (B) RH350, (C) RH500, and (D) RH800 biochar samples evaluated in this study. The resulting coefficient values are presented on the figures and summary of the statistical assessments are given in Table 3.


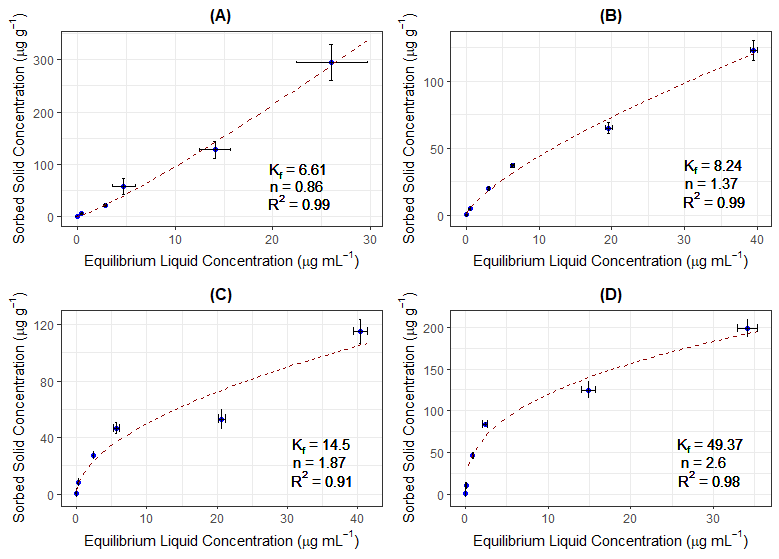


**Figure S10** Example of non-linear fits of the Freundlich isotherm with the solid concentration at equilibrium (Q_e_) and the resulting liquid concentration (C_e_) from each of the (A) wood pellet feedstock, (B) WP350, (C) WP500, and (D) WP800 biochar samples evaluated in this study. The resulting coefficient values are presented on the figures and summary of the statistical assessments are given in Table 3.


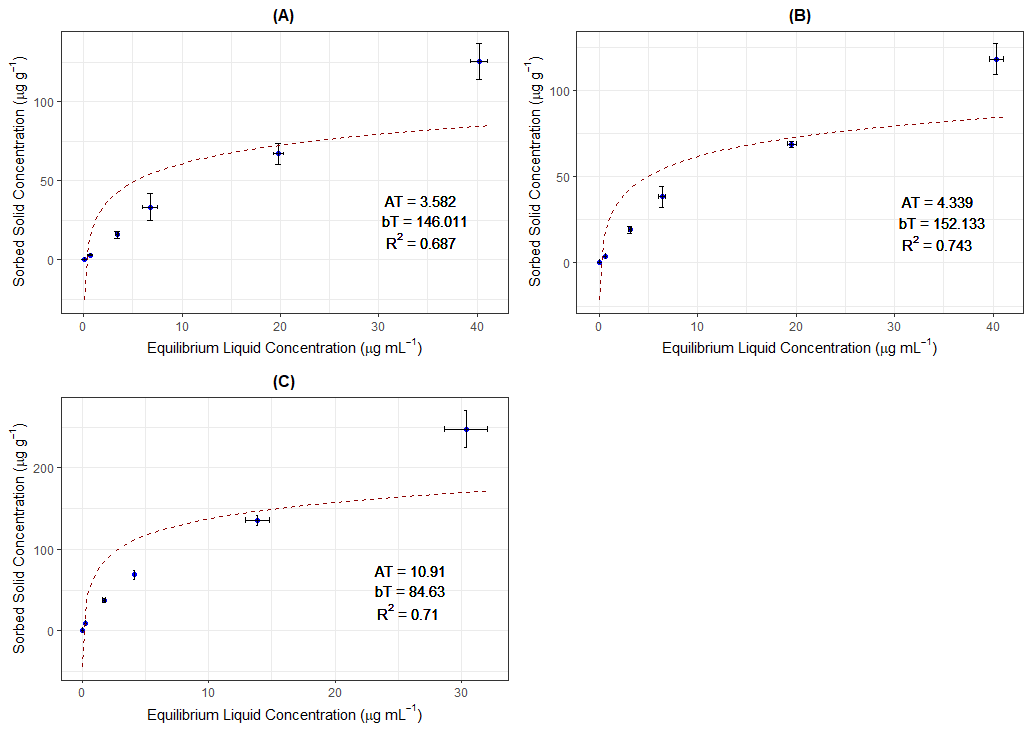


**Figure S11**. Example of non-linear fits of the Temkin isotherm with the solid concentration at equilibrium (Q_e_) and the resulting liquid concentration (C_e_) from each of the (A) poultry litter feedstock, (B) PM350, and (C) PM500 evaluated in this study. The PM800 did not fit the Temkin isotherm. The resulting coefficient values are presented on the figures and summary of the statistical assessments are given in Table 3.


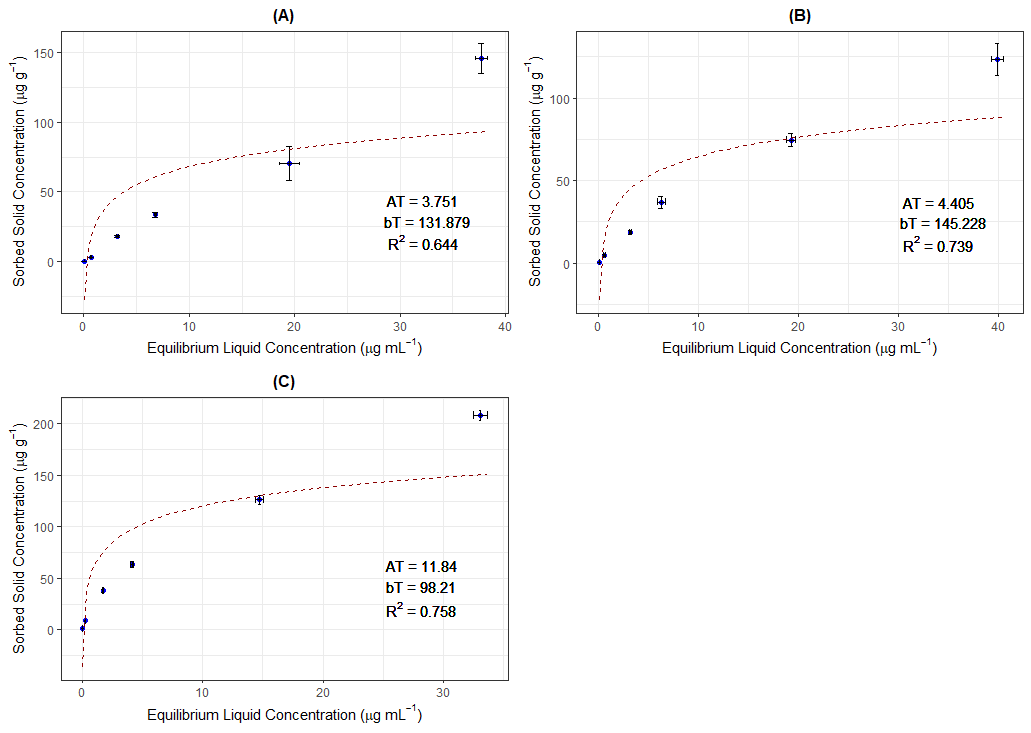


**Figure S12**. Example of non-linear fits of the Temkin isotherm with the solid concentration at equilibrium (Q_e_) and the resulting liquid concentration (C_e_) from each of the (A) rice hull feedstock, (B) RH350, and (C) RH500 biochar samples evaluated in this study. The RH800 did not fit the Temkin isotherm. The resulting coefficient values are presented on the figures and summary of the statistical assessments are given in Table 3.


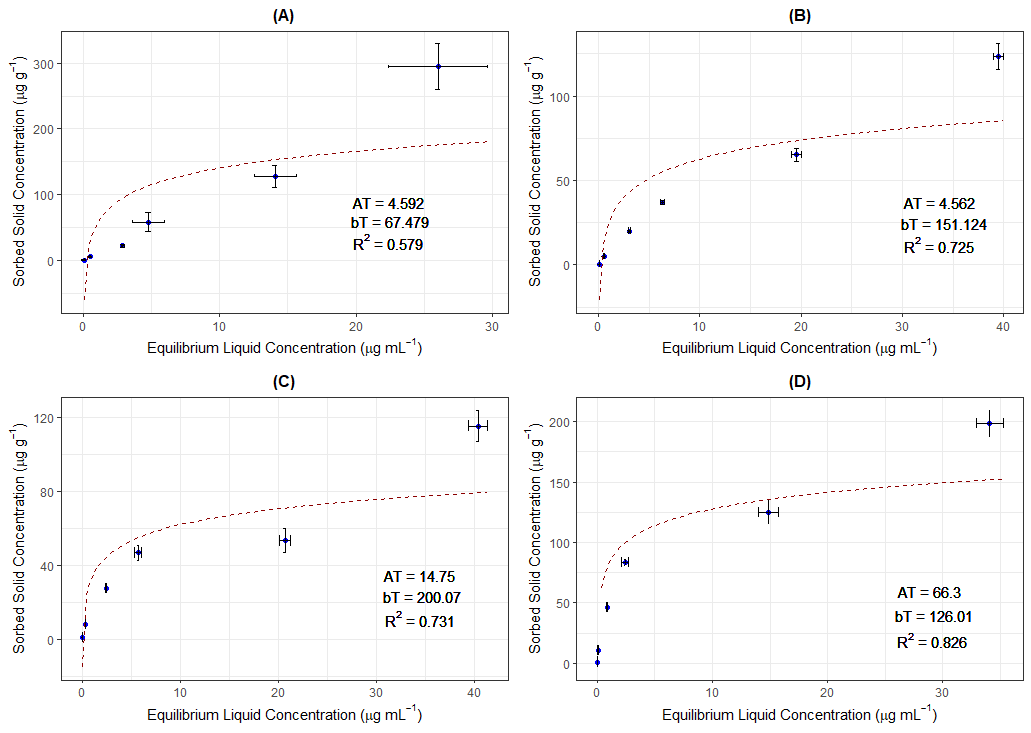


**Figure S13** Example of non-linear fits of the Temkin isotherm with the solid concentration at equilibrium (Q_e_) and the resulting liquid concentration (C_e_) from each of the (A) wood pellet feedstock, (B) WP350, (C) WP500, and (D) WP800 biochar samples evaluated in this study. The resulting coefficient values are presented on the figures and summary of the statistical assessments are given in Table 3.

**
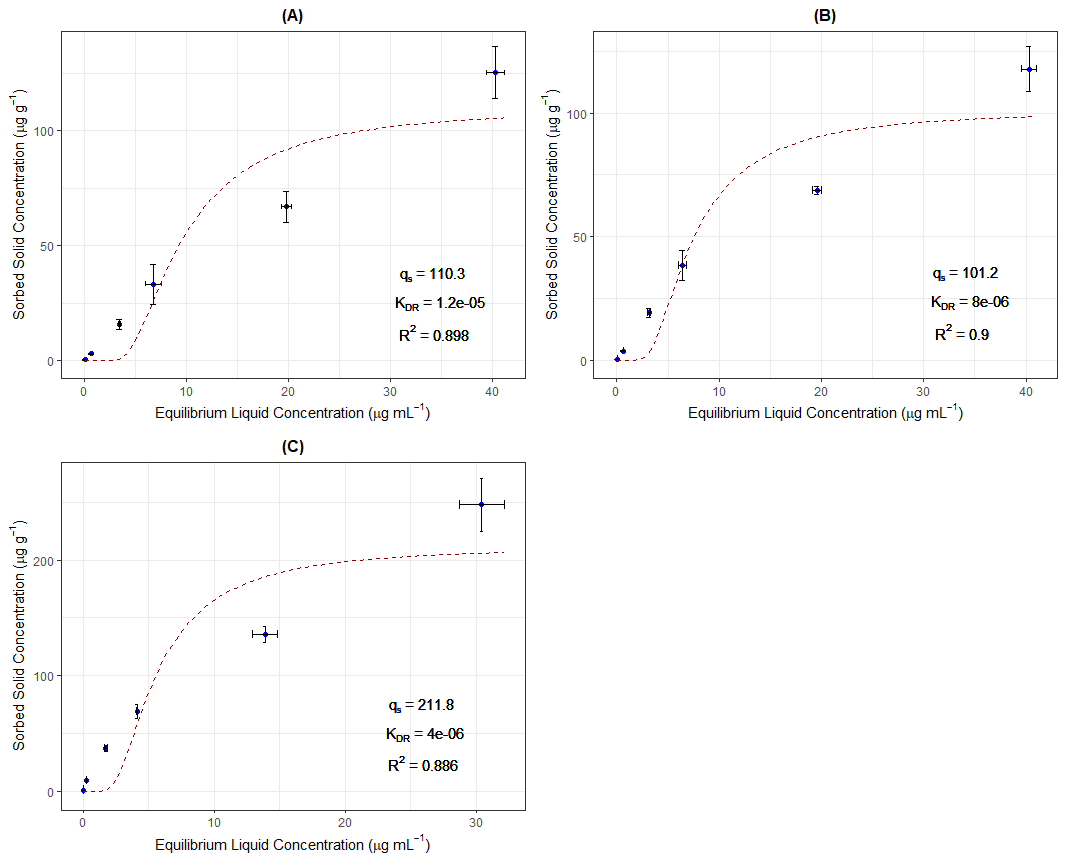
**

**Figure S14**. Example of non-linear fits of the Dubinin–Radushkevich isotherm with the solid concentration at equilibrium (Q_e_) and the resulting liquid concentration (C_e_) from each of the (A) poultry litter feedstock, (B) PM350, and (C) PM500 evaluated in this study. The PM800 did not fit the Dubinin–Radushkevich isotherm. The resulting coefficient values are presented on the figures and summary of the statistical assessments are given in Table 3.


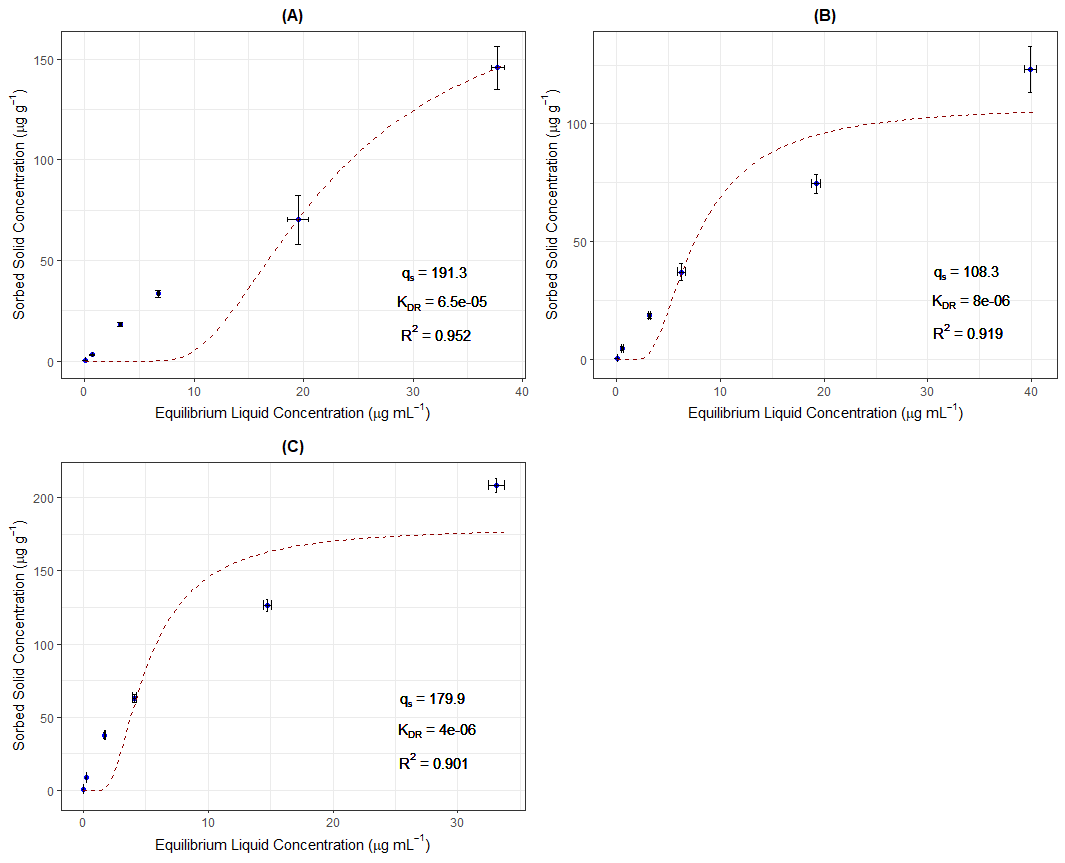


**Figure S15**. Example of non-linear fits of the Dubinin–Radushkevich isotherm with the solid concentration at equilibrium (Q_e_) and the resulting liquid concentration (C_e_) from each of the (A) rice hull feedstock, (B) RH350, and (C) RH500 biochar samples evaluated in this study. The RH800 did not fit the Dubinin–Radushkevich isotherm. The resulting coefficient values are presented on the figures and summary of the statistical assessments are given in Table 3.


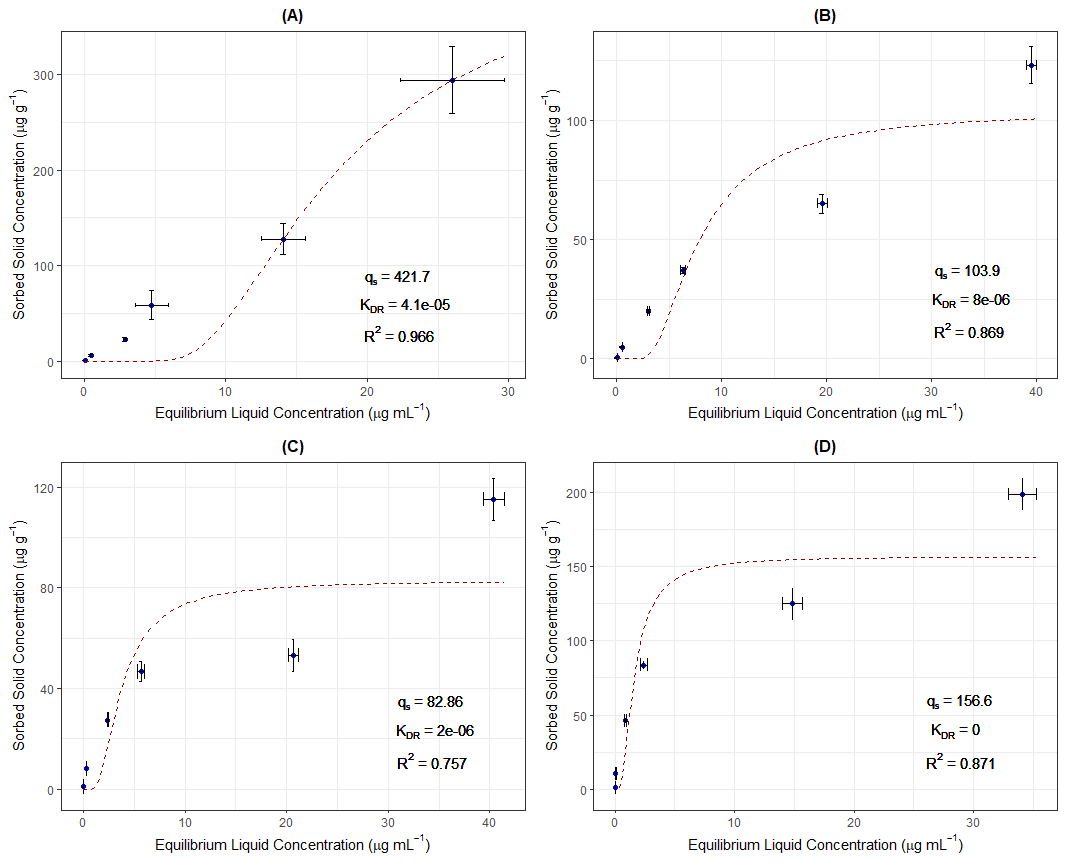


**Figure S16.** Example of non-linear fits of the Dubinin–Radushkevich isotherm with the solid concentration at equilibrium (Q_e_) and the resulting liquid concentration (C_e_) from each of the (A) wood pellet feedstock, (B) WP350, (C) WP500, and (D) WP800 biochar samples evaluated in this study. The resulting coefficient values are presented on the figures and summary of the statistical assessments are given in Table 3.


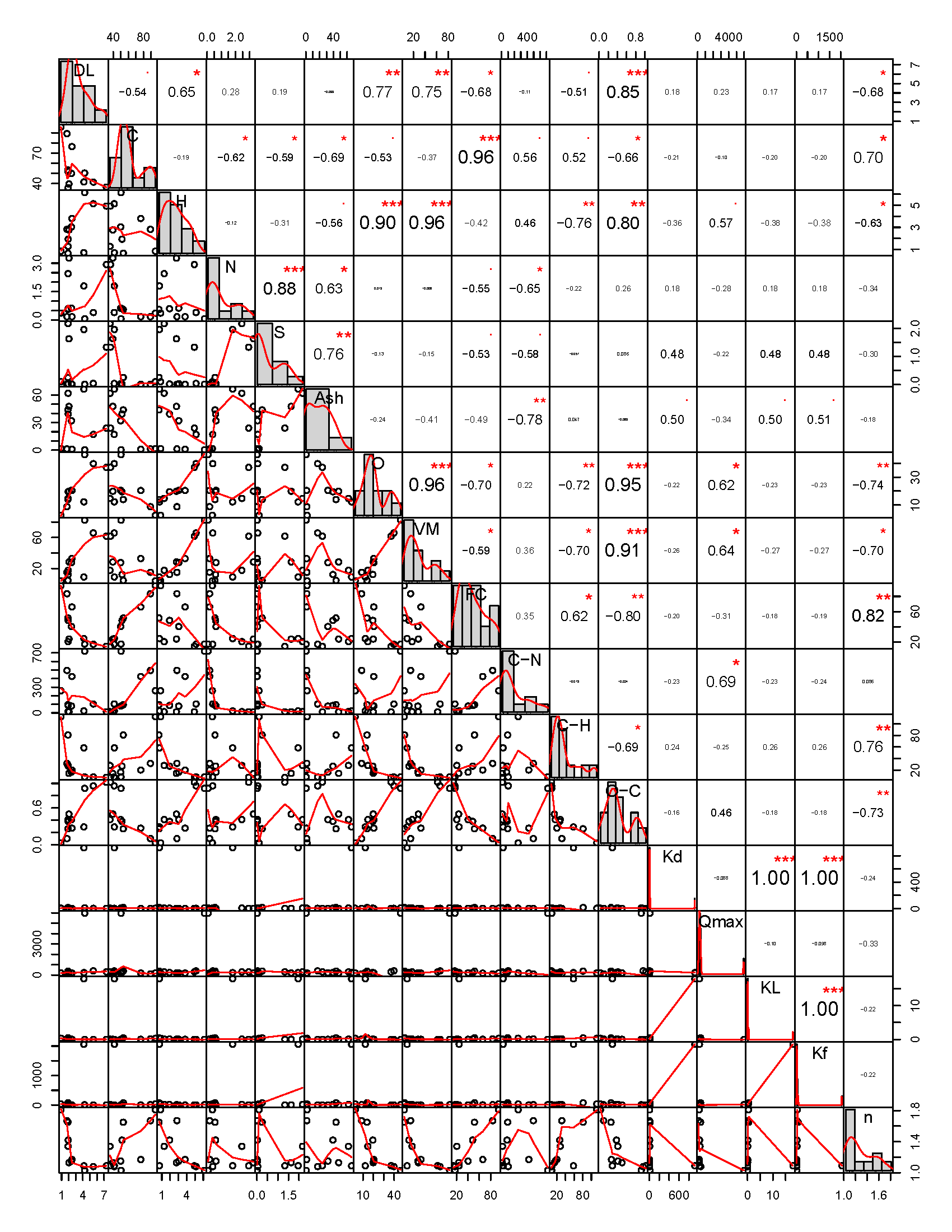


**Figure S17.** Correlation matrix for sorption coefficients and biochar properties.

**Table S1.** Details of liquid additions for each concentration level evaluated.

| **Sr. No.** | **0.01M CaCl_2_ added**  **(mL)** | **Cold Solution** | | **Radiolabeled Solution** | | **Total Volume (mL)** | **Initial Concentration (ppm)** |
| --- | --- | --- | --- | --- | --- | --- | --- |
|  |  | **Concentration (ppm)** | **mL Added** | **Concentration (ppm)** | **mL Added** |  |  |
| 1 | 2.9 | 3 | 0.1 | 0.18 | 0.2 | 3.2 | **0.1** |
| 2 | 2 | 3 | 1 | 0.18 | 0.2 | 3.2 | **0.9** |
| 3 | 2 | 15 | 1 | 0.18 | 0.2 | 3.2 | **4.7** |
| 4 | 1 | 15 | 2 | 0.18 | 0.2 | 3.2 | **9.4** |
| 5 | 2.2 | 100 | 0.8 | 0.18 | 0.2 | 3.2 | **25** |
| 6 | 1.4 | 100 | 1.6 | 0.18 | 0.2 | 3.2 | **50** |

**Table S2:** Description of sorption model equations and linearized forms used with the PUPAIM package.

| Isotherm Model | Equation | Linearized Form |
| --- | --- | --- |
| Simple Linear | $C_{s}=K_{D}C_{e}$ | $C_{s}=K_{D}C_{e}$ |
| Langmuir | $C_{s}=\frac{{Q_{o}bC}_{e}}{1+bC_{e}}$ | $\frac{C_{e}}{C_{s}}=\frac{1}{bQ_{o}}+\frac{C_{e}}{Q_{o}}$ |
| Freundlich | $C_{s}=K_{f}{C_{e}}^{\frac{1}{n}}$ | log($C_{s})=\log K_{f}+\frac{1}{n}log(C_{e})$ |
| Dubinin-Radushkevich | $C_{s}=Q_{DR}e^{-K_{DR}\varepsilon^{2}}$ | $ln(C_{s})={ln(C}_{e})-{K_{ads}\varepsilon^{2}}$ |
| Temkin | $C_{s}={\frac{RT}{b_{T}}ln(A_{T}C}_{e})$ | $C_{s}={\frac{RT}{b_{T}}\ln\left( A_{T} \right)+\frac{RT}{b_{T}}ln(C}_{e})$ |

**Notes:** The definition of the variables in the above table are given below: $C_{e}$is the equilibrium liquid concentration (mg L^-1^), $C_{s}$is the equilibrium solid concentration (ug g^-1^), K_D_ is the linear sorption coefficient (L kg^-1^), Q_o_ and b are the Langmuir coefficients, K_f_ and $\frac{1}{n}$ are the Freundlich coefficients, the Dubinin-Radushkevich isotherm has $\varepsilon$ and K_ADS_ as the fitting coefficients with $\varepsilon=RT ln\left( 1+\frac{1}{C_{e}} \right)$, where R is the universal gas constant, T is the temperature (^o^K), log is the base-10 logarithm, ln is the natural logarithm, and finally A_T_ and b_T_ are the Temkin coefficients.

**Table S3.** Analysis of variance for the dependence of the feedstock and pyrolysis temperature on the resulted observed values of K_D_ for the entire experiment.

summary(model)


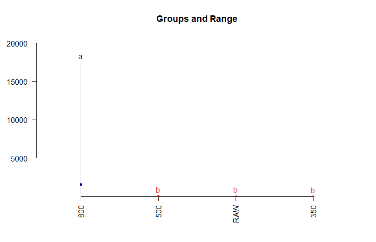
 Df Sum Sq Mean Sq F value Pr(>F)

Temp 3 99924894 33308298 19.83 2.54e-11 ***

Feedstock 2 54408267 27204133 16.20 2.96e-07 ***

Temp:Feedstock 6 163345939 27224323 16.21 3.12e-15 ***


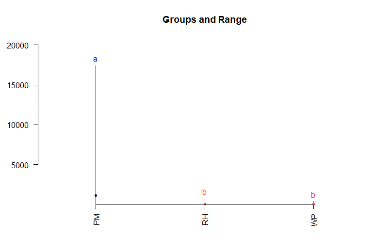
Residuals 204 342593564 1679380

---

Signif. codes: 0 ‘***’ 0.001 ‘**’ 0.01 ‘*’ 0.05 ‘.’ 0.1 ‘ ’ 1

Treatments with the same letter are not significantly different.

Kd groups


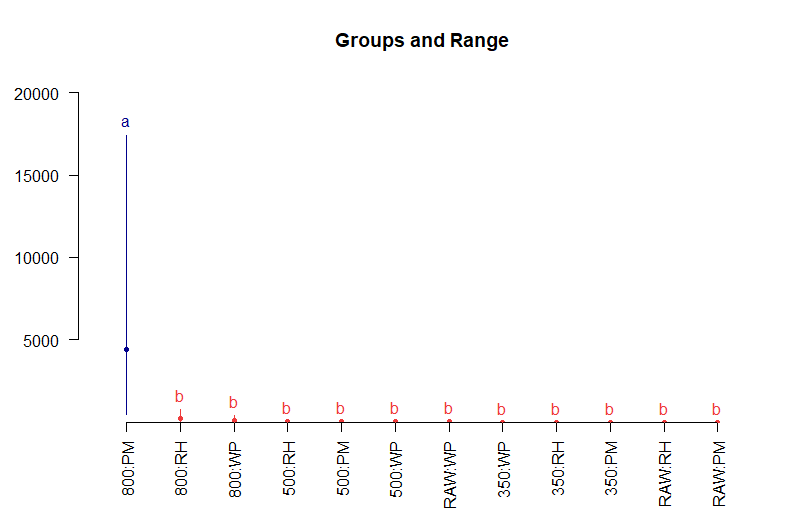
800:PM 4420.948351 a

800:RH 227.866150 b

800:WP 97.383446 b

500:RH 24.716040 b

500:PM 23.773192 b

500:WP 16.995001 b

RAW:WP 11.008526 b

350:WP 5.872060 b

350:RH 5.664629 b

350:PM 5.197731 b

RAW:RH 4.812454 b

RAW:PM 4.358673 b

**Table S4:** Percentage of MCPA desorption from studied biochars

| **Biochars** | **(0.1 µg mL^-1^)** | | **(0.9 µg mL^-1^)** | | **(4.7 µg mL^-1^)** | | **(9.4 µg mL^-1^)** | | **(25 µg mL^-1^)** | | **(50 µg mL^-1^)** | |
| --- | --- | --- | --- | --- | --- | --- | --- | --- | --- | --- | --- | --- |
|  | **Avg.** | **SE** | **Avg.** | **SE** | **Avg.** | **SE** | **Avg.** | **SE** | **Avg.** | **SE** | **Avg.** | **SE** |
| **PM_Raw_** | 49.54 | 2.01 | 47.32 | 3.04 | 54.57 | 4.89 | 62.79 | 3.83 | 38.98 | 2.07 | 42.11 | 1.72 |
| **PM350** | 40.21 | 1.30 | 41.64 | 2.03 | 50.77 | 2.97 | 51.36 | 4.23 | 31.29 | 1.60 | 31.58 | 9.61 |
| **PM500** | 11.83 | 0.61 | 10.02 | 0.83 | 17.17 | 0.81 | 22.78 | 0.67 | 18.89 | 1.06 | 31.14 | 1.82 |
| **PM800** | -0.23 | 0.31 | 0.02 | 0.11 | 0.03 | 0.08 | -1.48 | 0.62 | -0.12 | 0.12 | -0.07 | 0.26 |
| **RH_Raw_** | 50.89 | 3.57 | 77.52 | 17.90 | 60.94 | 2.75 | 67.94 | 2.12 | 47.80 | 4.40 | 47.70 | 2.55 |
| **RH350** | 40.89 | 2.67 | 50.28 | 2.69 | 50.14 | 2.62 | 54.61 | 4.27 | 44.41 | 2.15 | 37.33 | 1.86 |
| **RH500** | 10.11 | 0.43 | 7.30 | 2.54 | 8.90 | 0.99 | 12.98 | 2.28 | 8.82 | 0.66 | 15.98 | 2.96 |
| **RH800** | -0.24 | 0.20 | -1.18 | 0.29 | -4.81 | 3.20 | -1.08 | 0.74 | -5.00 | 0.68 | -4.47 | 1.12 |
| **WP_Raw_** | 42.35 | 4.57 | 41.82 | 1.06 | 52.80 | 2.72 | 53.42 | 5.81 | 39.41 | 2.22 | 37.78 | 2.73 |
| **WP350** | 42.56 | 2.49 | 34.48 | 2.90 | 56.10 | 15.29 | 43.92 | 0.61 | 37.60 | 4.60 | 43.45 | 0.75 |
| **WP500** | 9.89 | 0.40 | 12.54 | 0.32 | 21.96 | 3.74 | 33.72 | 2.94 | 56.88 | 7.60 | 41.23 | 5.26 |
| **WP800** | 1.76 | 0.65 | 1.12 | 0.52 | 3.26 | 1.54 | 12.77 | 2.33 | 14.19 | 2.51 | 35.77 | 5.68 |

**Table S5:** ANOVA analysis for sorption coefficient (K_D_) and the influence of feedstock and pyrolysis temperature.

ANOVA summary for desorption KD :

model<-aov(data=mcpa,Kd_desorb~Feedstock*Temp)

> summary(model)

Df Sum Sq Mean Sq F value Pr(>F)

Feedstock 2 2.826e+08 141278972 11.45 1.94e-05 ***

Temp 3 4.962e+08 165393212 13.40 5.09e-08 ***

Feedstock:Temp 6 8.477e+08 141288499 11.45 4.98e-11 ***

Residuals 204 2.517e+09 12338262

---

Signif. codes: 0 ‘***’ 0.001 ‘**’ 0.01 ‘*’ 0.05 ‘.’ 0.1 ‘ ’ 1

> out<-HSD.test(model,c("Temp"), group=TRUE,console=TRUE)

Study: model ~ c("Temp")

HSD Test for Kd_desorb

Mean Square Error: 12338262

Temp, means

Kd_desorb std r Min Max

350 5.167234 1.783517 54 1.2241642 11.11902

500 29.077764 22.051582 54 1.4111889 76.43220

800 3513.288163 8295.563758 54 4.9605828 51289.20000

RAW 5.258299 3.047970 54 0.7898116 14.10653

Alpha: 0.05 ; DF Error: 204

Critical Value of Studentized Range: 3.663283

Minimun Significant Difference: 1751.058

**Table S5. (Continued)**

Treatments with the same letter are not significantly different.

Kd_desorb groups

800 3513.288163 a

500 29.077764 b

RAW 5.258299 b

350 5.167234 b

> out<-HSD.test(model,c("Feedstock"), group=TRUE,console=TRUE)

Study: model ~ c("Feedstock")

HSD Test for Kd_desorb

Mean Square Error: 12338262

Feedstock, means

Kd_desorb std r Min Max

PM 2505.25604 7367.6859 72 2.4818282 51289.2000

RH 112.07401 289.4394 72 0.7898116 1438.6962

WP 47.26355 111.9381 72 1.2241642 617.9444

Alpha: 0.05 ; DF Error: 204

Critical Value of Studentized Range: 3.338885

Minimun Significant Difference: 1382.172

Treatments with the same letter are not significantly different.

Kd_desorb groups

PM 2505.25604 a

RH 112.07401 b

WP 47.26355 b
